# Polyphenols, aging, and health: What can we expect from the food industry in the technology era?

**DOI:** 10.3389/fmed.2025.1671886

**Published:** 2025-11-06

**Authors:** Iramaia Angelica Neri Numa, Renata Aparecida Soriano Sancho, Kendji Eduardo Wolf, Cynthia Tereza Corrêa da Silva Miranda, Stephanie Dias Soares, Adriana de Souza Lima, Glaucia Maria Pastore

**Affiliations:** 1 Laboratory of Bioflavours and Bioactive Compounds, Department of Food Science and Nutrition, Faculty of Food Engineering, University of Campinas, Campinas, Brazil; 2 Polytechnic School of the University of São Paulo - PECE - Poli-USP Continuing Education Program, São Paulo, Brazil; 3 SEDA Executive Education, São Paulo, Brazil; 4 Neoway, uma empresa B3, São Paulo, Brazil; 5 Faculty of Pharmaceutical Sciences, Federal University of Amazonas (UFAM), Manaus, Brazil

**Keywords:** polyphenols, aging, health, food industry, technology era

## Abstract

**Background:**

Aging is a physiological process characterized by several metabolic changes, oxidative stress and inflammatory processes accumulated throughout life, reflecting in several diseases. Adopting a healthy lifestyle, including a diet rich in polyphenols, can play a crucial role in promoting healthy aging, since these compounds are capable of regulating several signaling pathways as well as modulating the intestinal microbiota and being involved in epigenetic modifications. With the advancement of technology and omics sciences, the food industry is undergoing a rapid transformation toward personalizing and customizing nutritional solutions that consider each individual’s health conditions, preferences, and lifestyles. Therefore, such niche that will become predominant worldwide.

**Scope and approach:**

The scope of this review focuses on the role of polyphenols in diseases related to aging, providing an overview of alternatives for personalized nutritional plans, including the role of the food industry in the technological era to meet the unique needs and to develop new food products for the elderly population.

**Key findings and conclusion:**

Food products for older people represent opportunities in a niche that will become predominant worldwide. In this sense new technologies offers new possibilities for innovation in the food industry production chain that aim to improve not only the nutritional characteristics of foods but also improvements in food processing, food safety, and quality assurance.

## 1 Introduction

Over the past few decades, we have witnessed a progressive increase in population aging worldwide due to an epidemiological transition process. It reflects not only in the life of the elderly individual but also in the economic, social, political, and health sectors (1, 2). According to the United Nations in its latest technical report, “World Population Ageing 2019: Highlights,” prepared by the Department of Economic and Social Affairs, by 2050, 1 in 6 people in the world will be over the age of 65 (2). Faced with the reality of these numbers, we must think not when we will grow old, but how we will grow old. Although deterioration in physical function cannot be prevented, successful aging is perfectly possible when all age groups are taken into account and preventive health measures are taken to maintain quality of life and wellbeing, thus reducing expenses with potential health problems arising from old age (1).

Emerging evidence has suggested that dietary interventions exert positive effects on human aging as diets rich in fruits, vegetables, whole grains, legumes, oils, and nuts appear capable of preventing and/or ameliorating age-associated dysfunctions including metabolic syndromes, cardiovascular diseases, and neurodegenerative disorder. The mechanism for these beneficial effects resides in the interaction with bioactive constituents found in various natural sources such as plant, microbial, and aquatic cyanobacteria. Their structures are categorized into phenolic compounds, terpenoids, alkaloids, nitrogen-containing compounds, organosulfur compounds, MUFA (Monounsaturated Fatty Acids), PUFA (Polyunsatured Fatty Acids), etc., (3).

Most of such phytochemicals support health by reducing oxidative stress and inflammation, enhancing cellular defenses, and modulating gene expression. As result, lowering risks of chronic diseases, including cardiovascular ones and cancer (4). Particularly, phenolic compounds improve insulin sensitivity, help control glycemia and weight gain, and provide cardio– and neuroprotection, largely through their antioxidant, anti-inflammatory, and immunomodulatory effects at both intestinal and systemic level (5). It means that gut microbiota can catabolize polyphenols by deglycosylation and that release of metabolites may exert additional health effects on the gut or be absorbed and further metabolized by phase II metabolism, up-regulating protein related to defensive mechanisms or metabolizing enzymes (5).

However, a better understanding of the role of polyphenols in the modulation of gut ecology, including the underlying mechanisms and beneficial effects in older subjects, is still necessary. Thus, the present review aims to provide up-to-date data on the role of polyphenols against chronic diseases and their contribution to redox modulation in old age, which helps to consider them in nutritional interventions for future clinical settings in preventing and treating aging-related diseases.

We also cannot fail to mention that “omics” studies integrated with big data technologies have changed the way of understanding the biological potential of bioactive compounds, becoming important tools for studying food-gene interactions, generating mechanistic insights, and informing the evolution of personalized nutrition. Therefore, the themes will be approached in a simplified way, citing some examples in the following sections.

## 2 Dietary polyphenols as antioxidants and their relationship with gut microbiota and aging-related diseases: what do we know so far?

According to the World Health Organization in its “World Report on Ageing and Health,” the changes that constitute and influence aging are complex. At a biological level, aging is associated with the gradual accumulation of various molecular and cellular damage. Over time, this damage leads to a gradual decrease in physiological reserves, an increased risk of many diseases, and a general decline in the individual’s capacity. Ultimately, it will result in death (6).

**FIGURE 1:**
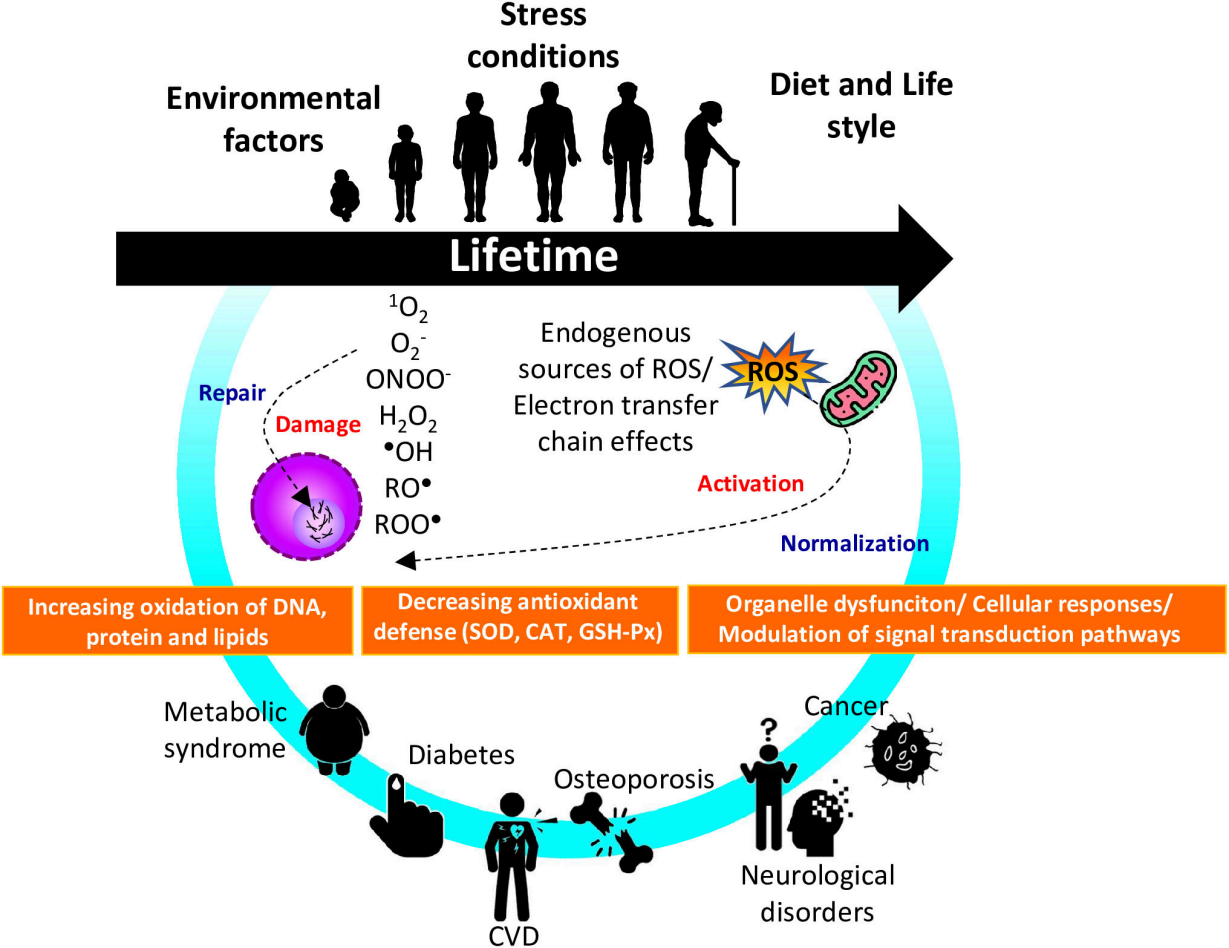
Schematic representation on oxidative and nitrosative strees involved in aging process and aging-related diseases. Reactive species are molecules with unpaired electrons derived from oxygen or nitrogen (ROS or RNS). They are produced endogenously through mitochondrial metabolism and in response to external factors such as xenobiotics, inflammation, toxins, or radiation. Antioxidant mechanims, enzymatic or non-enzymatic, normally regulate ROS/RNS levels to maintain physiological balance. When their production exceeds the organism’s defense capacity, oxidative strees occurs, leading to damage of DNA, proteins, lipids and carbohydrates. This imbalance can activate inflammtory pathways (e.g., NF-κB) and contribute to cellular senescence, mitochondrial dysfunction, and altered microRNA expression, processes linked to aging and chronic diseases such as metabolic syndrome, CVD, osteoporosis, neurodegenerative disorders, and cancer. ^1^O_2_, singlet oxygen; O_2_^∙–^ superoxide anion; H_2_O_2_, hydrogen peroxide; ^∙^OH, hydroxil radical; RO^∙^, alkoxyl radical; ROO^∙^, peroxyl radical. Source: (1–3).

Under normal physiological conditions, ROS/RNS result from metabolic processes; they are essential for several cellular mechanisms, including redox homeostasis, where the rates and amplitudes of generation and elimination of these reactive species are controlled. However, when redox homeostasis is disturbed, and there is an overproduction of pro-antioxidant agents, organisms lose the ability to detoxify reactive intermediates. Non-detoxified reactive species containing one or more unpaired electrons are more reactive and are involved in cellular dysfunctions. Impairment of the gut microbiota is triggered in addition to various molecular mechanisms that will lead to protein denaturation and enzyme inactivation, as well as mutations, genetic instability, and epigenetic modifications (Figure 1) (7).

**FIGURE 2:**
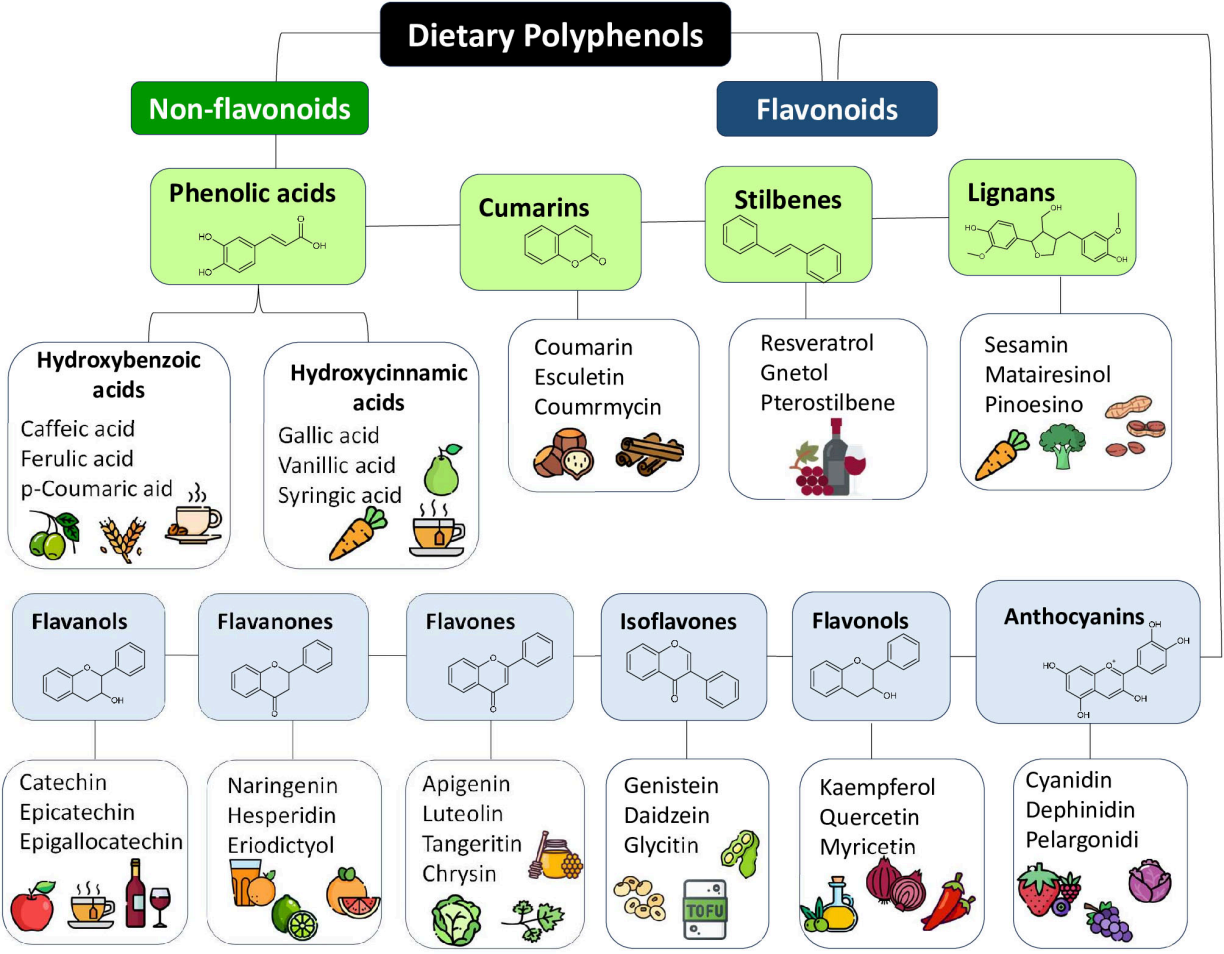
Dietary polyphenols classification, their basich chemical structure and some representative typical sources. Polyphenols are a diverse group of compounds characterized by the presence of one or more hydroxyl groups attached to aromatic rings. They are naturally found in a wide variety of plant-derived sources, including fruits, vegetables, nuts, whole grains, olive oil, tea, wine, flowers, and tree barks. Source: (4, 5).

In this scenario, polyphenols draw attention due to their chemical and physical properties since they can act as antioxidant and/or pro-oxidant properties, depending on their related structure (e.g., flavonoids, stilbenes, lignans, and phenolic acids) and/or the cellular redox context, which may include increased levels of oxidant-eliminating proteins or reduced levels of oxidized proteins and lipids, acting in various signaling pathways (8) (Figure 2).

Although polyphenols are readily ionized (owing to their proneness for electronic delocalization), their bioactivity relies heavily on the position of hydroxyl groups and relative ease of substituent modification since a large number of methylations will exhibit less antioxidant (8). Also, polyphenols are restricted in their antioxidant capacity and interaction with proteins involved in the transcription and expression of genes related to metabolism, proliferation, inflammation, and growth (9).

There is much talk about the biological properties of dietary polyphenols, but the fact is that they will only be effective if they are bioavailable and are absorbed and metabolized in the gastrointestinal tract (GIT). The first thing we need to understand is that the bioavailability of polyphenols requires that these compounds be released from the food matrix during digestion. Once bioavailable, their metabolites are absorbed through the intestinal epithelium and distributed to peripheral tissues, where they can exert functional effects (10, 11). Importantly, most polyphenols undergo extensive metabolic transformation in the GIT, largely mediated by the intestinal microbiota. This microbial metabolism not only facilitates their conversion into bioactive forms but also contributes to shaping microbial community composition and diversity (10).

Polyphenols are increasingly recognized as prebiotic-like agents, influencing gut microbiota balance and short-chain fatty acid (SCFA) production. SCFAs are efficiently absorbed in the colon and serve multiple physiological roles, including supporting epithelial barrier integrity, regulating cellular growth, and modulating immune responses (11). The gut microbiome thus operates as a key metabolic hub, with activities spanning catabolism, bioconversion, and synthesis of diverse compounds that can affect both local and systemic physiology. The advent of high-throughput meta-omics technologies has further highlighted the gastrointestinal tract; home to the densest microbial communities, dominated by Firmicutes, Bacteroidetes, and Actinobacteria as a central site of these host-microbe interactions (11).

**FIGURE 3:**
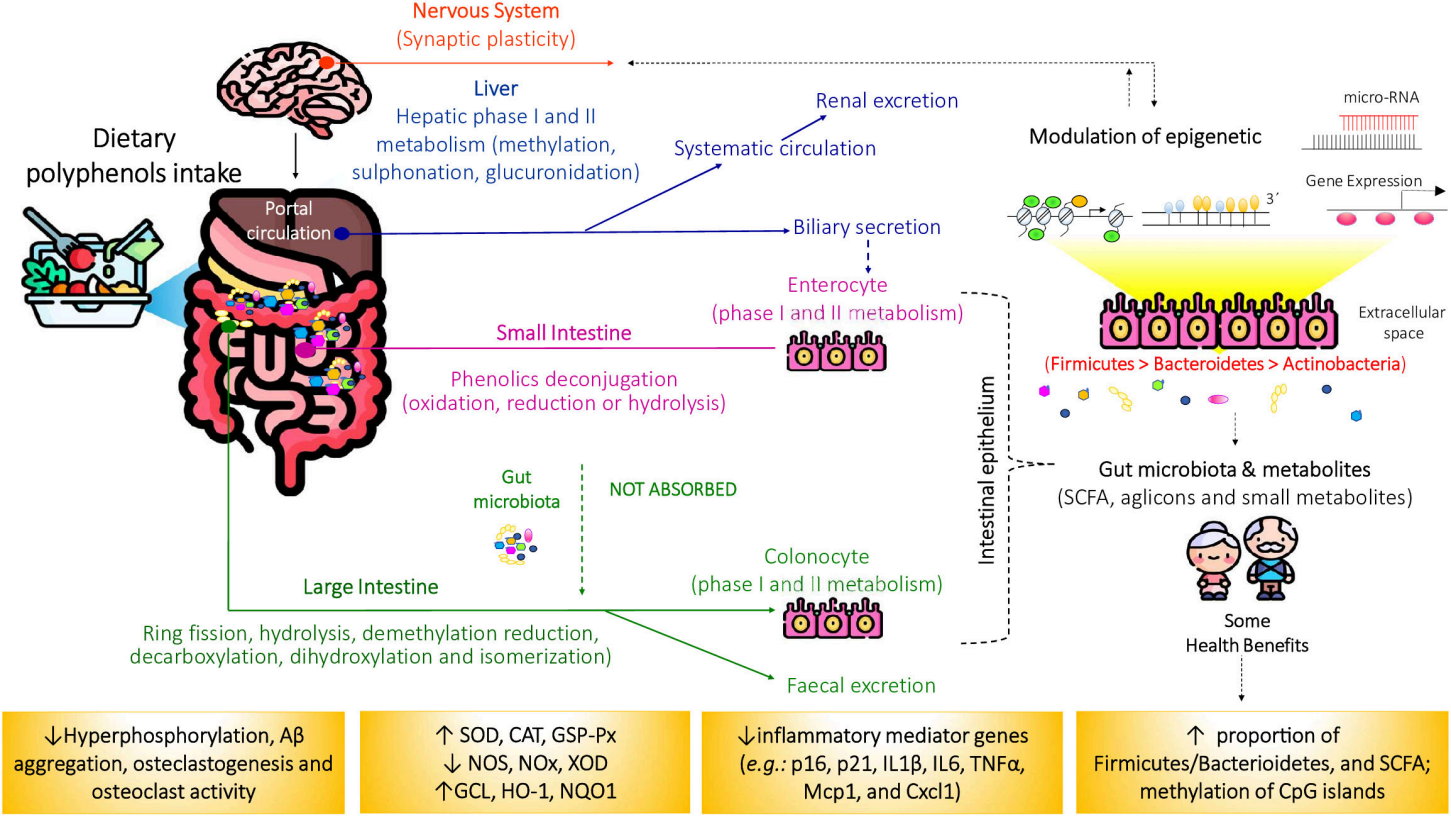
Schematic representation of dietary polyphenols and microbial-derived metabolites on aiging-related diseases. Only a small fraction (5%–10%) of dietary polyphenols is absorbed in the small intestine. Upon reaching the colon, the remaining compounds are metabolized by gut microbiota, producing short-chain fatty acids (SCFAs) and other polyphenol-derived metabolites through various biochemical pathways. These metabolites exert beneficial effects by modulating inflammation, inducing apoptosis, preventing obesity, insulin resistance, and oxidative stress, as well as influencing gene expression and epigenetic regulation. CAT, catalase; GCL, animal glutamate cysteine ligase; DNMT, DNA methyltransferase; GSH, glutathione peroxidase; GSH-Px, glutathione reductase; HAT, histone acetylases; HDAC, histone deacetylases; NOS, nitric oxide synthase; NOX, nicotinamide adenine dinucleotide phosphate oxidase (NOX); NQO1, NAD(P)H quinone acceptor oxidoreductases; SOD, superoxide dismutase; XOD, xanthine oxidase. Source: (6, 7).

However, microbiota composition is not static. Numerous intrinsic and extrinsic factors, such as age, diet, lifestyle, and medication, can disrupt microbial homeostasis, leading to dysbiosis. This imbalance impairs host regulatory pathways and predisposes individuals to disease (11). The gastrointestinal tract is particularly vulnerable to age-associated changes, including reduced barrier function, impaired nutrient assimilation, and a higher risk of chronic disease. In older adults, gut microbiota diversity typically declines, with beneficial taxa decreasing and facultative anaerobes increasing, which is often accompanied by reduced SCFA production (12). The main interactions between the changes in the gut microbiota in older people are summarized in Figure 3 and Supplementary Material 1.

Dietary inputs are thus critical in shaping microbial diversity and metabolic output. Microbiota-derived metabolites can be regarded as terminal products of microbial gene expression and metabolic activity, forming a functional readout of host–microbe interactions. Once that microbiota species is interchangeable in terms of functions using the metabolites produced by the action of gene products contained in the gut bacteria (13). Metabolites produced by the action of microbiota are the downstream product of gene expression and metabolic activity, and, therefore, they can be considered a final output within the functional hierarchy. This perspective aligns with the rise of nutrigenomics, which investigates how diet influences gene expression via epigenetic regulation, transcriptomic shifts, and microbiome modulation (14). In parallel, biogerontology emphasizes the interplay between nutrition, genetics, chronic disease, and longevity, underscoring nutrition as a strategy to promote healthy aging (13).

Evidence suggests that maintaining a diverse and balanced microbiota is a hallmark of healthy aging, whereas dysbiosis is associated with frailty and multimorbidity (15). Concepts such as resistance (the ability to withstand perturbations), resilience (capacity to recover initial composition after disturbance), and functional redundancy (maintenance of function despite compositional shifts) are central to understanding how microbiota adapt over time. These properties are influenced by age, geography, lifestyle, and medical interventions (13).

Recent clinical and experimental findings indicate that polyphenol-rich interventions can beneficially modulate microbial communities (16). For instance, a randomized crossover trial demonstrated that cocoa flavanols increased populations of Bifidobacterium and Lactobacillus while reducing *Clostridium* spp., changes that were accompanied by significant decreases in plasma triacylglycerols and C-reactive protein. Such results reinforce the notion that polyphenol-derived prebiotics can regulate pathogenic and commensal bacteria, strengthen host–microbe interactions, and ultimately contribute to improved health outcomes (17).

Similarly, it was found that the administration of syringaresinol modulated the gut microbiota by increasing the relative abundance of the *Lactobacillus* and *Bifidobacterium* species while decreasing levels of the potentially harmful *Akkermansia* genus. It, in turn, effectively improved age-related dysregulation of mouse lymphocyte subsets (18). Also, the procyanidin B2 may significantly ameliorate the cognitive decline and oxidative damage in mice induced by D-galactose since the relative percent of *Roseburia, Lachnospiraceae*, and *Bacteroides* was increased considerably in D-galactose mice treated with procyanidin B2, which had the advantage to produce butyrate as the main compound (19).

Likewise, both quercetin and the glycosylated forms of quercetin, myricetin, and kaempferol can simultaneously induce the growth of *Akkermansia muciniphila* and reduction of the *Firmicutes/Bacteriodetes* ratios. Both are associated with reduced body weight, oxidative stress, intestinal and hepatic inflammation, and improved insulin sensitivity upon diets rich in fat and sucrose (20). Also, the 4,4′- dimethoxychalchone, epigallocatechin gallate, silibinin, and pelargonidin-3-O-glucoside can attenuate hyperglycemia by inducing autophagy and modifying gut microbiota composition by an increased abundance of *Prevotella*, and elevated *Firmicutes/Bacteriodetes* ratio besides to strengthen the intestinal barrier integrity (21).

Given that, we should consider three points for highlighting: (1) New solutions are imperative to mitigate age-related health problems; (2) Decreases in microbial diversity are considered an indicator of an unhealthy microbiome since it is associated with different chronic conditions in older people; (3) Data on the impact of gut microbiota on flavonoids metabolites and their mechanisms of actions to promote resilience in elderly microbiota are still scarce. Therefore, the next section will discuss the specific cause-and-effect relationships between microbial dynamics and polyphenols intake and the mechanistic basis of these relationships using *in vitro in vivo* age models to establish intestinal biomarkers of human aging.

## 3 Dietary polyphenols in managing aging-related diseases

### 3.1 Type 2 diabetes

Type 2 Diabetes Mellitus (Type 2 DM) is characterized by chronic hyperglycemia triggered by: - pancreatic β-cell dysfunction that reduces insulin secretion, - insulin resistance leads to increased hepatic glucose production and reduced glucose uptake in liver, muscle, and adipose tissue (22). It may represent a pro-aging state since age-related comorbidities, such as frailty, mild cognitive impairment, Alzheimer’s disease, cardiovascular disease, visual impairment, and renal dysfunction, are more common in diabetic individuals than in non-diabetics (23). This may occur, for example, due to increased oxidative stress and chronic inflammation, which are common in hyperglycemic states. Persistent hyperglycemia, a hallmark of diabetes, increases ROS generation (or by altering the redox balance) and oxidative stress markers, with an accompanying decrease in antioxidant levels (24).

Numerous well-established pathways are involved, such as enhanced intracellular formation of advanced glycation end products, elevated flux in the polyol pathway, activation of protein kinase C, or excessive superoxide production by the mitochondrial electron transport chain (25). Another mechanism involved in the cellular response to oxidative stress is poly(ADP-ribosyl)ation (PARylation), a post-translational process involving the addition of ADP-ribose polymers to target proteins by poly(ADP-ribose) polymerases (PAR). Studies have shown that elevated PAR levels are linked to hyperglycemia and subsequent oxidative stress in various tissues and cells of diabetic individuals (26–28).

Epidemiological studies have shown that daily dietary intake influences the prevention and mitigation of comorbidities associated with type 2 DM, including processes related to aging. Polyphenol-rich diets, for example, may contribute to glycemic control through different mechanisms, such as reducing glucose absorption, protecting beta cells, increasing insulin secretion, reducing hepatic gluconeogenesis, and improving glucose uptake in peripheral tissues, in addition to the antioxidant effect (29).

A study evaluated the effect of Resveratrol (RV) supplementation in 124 older subjects (men and women) aged between 60 and 74 years and with type 2 DM in a double-blind randomized clinical trial for 6 months. As a result, it was observed a reduction in triglyceride levels, a lower oxidative stress index, and an increase in the concentration of sirtuin 1 (30).

In parallel, a single-blind, parallel-group, randomized controlled clinical trial consisting of a 6-month treatment period was conducted by Mahjabeen et al. (31) in 472 elderly patients with type 2 DM. Of these, 242 subjects received 500 mg/day of RV and had decreased levels of glycated hemoglobin/hemoglobin A1c, C-reactive protein, and pro-inflammatory cytokines (IL-6, TNF-α, and IL-1β) compared to placebo. RV administration also improved renal function by levels of glutamic oxaloacetic transaminase, serum glutamic pyruvic transaminase, alkaline phosphatase, albumin, blood urea nitrogen, and creatinine (31). Furthermore, a randomized, double-blind, placebo-controlled, parallel-group trial with diabetic patients (≥ 50 years old), 45 of whom received 200 mg/day of RV for 24 weeks. RV supplementation significantly reduced plasma glucose, insulin, homeostatic model assessment of insulin resistance, malondialdehyde, high-sensitivity C-reactive protein, tumor necrosis factor alpha, and interleukin-6. RV also modulated the expressions of circulating microribonucleic acids (miRNAs) 21 and 34a and, for the first time, was shown to decrease the expression of miRNA-375 significantly (31). miRNAs have been studied as therapeutic biochemical markers for diagnosing and treating type 2 DM (32). Additional studies, with supplementation of other phenolics, are shown in Supplementary Material 2.

### 3.2 Cardiovascular diseases

Cardiovascular diseases (CVD) is a general term describing a disease of the heart or blood vessels. Different CVD can affect humans, among them four main types: coronary heart disease, stroke, peripheral arterial disease, and aortic disease.

By the end of 2020, deaths from CVD represent 32% of all deaths globally, a reflection of the rapid epidemiologic transition, particularly in low-and middle-income countries. Although the net percentage of deaths caused by CVD overall has increased, this mainly reflects a rise in low and middle-income countries and a decline in high-income countries. CVD now causes most deaths in all low and middle-income regions and is the leading cause in those 50 years and older. Worldwide, CVD is largely driven by modifiable risk factors, such as smoking, lack of physical activity, and diets high in fat and salt. Elevated blood pressure and cholesterol levels are the leading causes, with tobacco, obesity, and physical inactivity remaining important contributors as well (33).

In addition to the factors already mentioned, aging and inflammation have a close relationship with the progression of CVD (34). Besides the involvement of the inflammatory process, the role of oxidative stress has also been evidenced in the development and progression of CVD. Elevated levels of various pro-inflammatory markers have been found in cardiac patients and correlated with the prognosis and severity of the disease, suggesting a direct relationship between inflammation markers and the risk of future cardiovascular events. Elevated levels of C-reactive protein and interleukin-6 (IL-6) were found in patients with unstable angina, and an early increase in pro-inflammatory cytokines such as TNF-alpha, IL-6, IL-1beta, and transforming growth factor 1-beta (TGF-1beta) were observed in response to acute myocardial infarction (35). In addition to cytokines, reactive oxygen species (ROS) also play a role in cardiovascular pathologies, as seen in chronic heart failure (36). Oxidative stress and ROS affect smooth muscle cells and endothelial cells, causing endothelial damage and developing atherosclerosis, which can lead to myocardial infarction and ischemic reperfusion (37).

Phenolic compounds present in the diet are known to exert beneficial effects on cardiovascular function. The cardioprotective role of polyphenols involves various mechanisms, including (1) increasing nitric oxide (NO) release and vasodilation in endothelial cells through various effects of endothelial nitric oxide synthase (eNOS); (2) modulating the nuclear factor erythroid-2-related factor 2 - antioxidant response element (Nrf2-ARE), promoting positive regulation of antioxidant enzyme expression; and (3) negatively regulating excessive generation of reactive oxygen species (ROS) and activation of nuclear factor kappa B (NF-κB), the main regulator of the inflammatory response that drives cytokine production (38). An example of this is resveratrol, a polyphenol belonging to the stilbene class, found in high concentrations in red wine, red grapes, berries, strawberries, raspberries, tomatoes, and some nuts (39).

Several studies have demonstrated the potential of resveratrol. In mice with left ventricular hypertrophy, preserved ejection fraction, diastolic dysfunction, and pulmonary congestion, resveratrol significantly reduced the release of IL-1β, IL-6, and TNF-α, as well as catalase (CAT), superoxide dismutase (SOD), and glutathione (GSH) activities (40). In a double-blind, randomized study involving 60 patients with heart failure with reduced ejection fraction, aged 66.7 ± 2.01 years, daily intake of 100 mg of resveratrol for 3 months resulted in decreased erythrocyte aggregation, which may contribute to improved coronary and peripheral blood flow in heart failure (36).

In addition to resveratrol, the cardioprotective effect has already been investigated in other polyphenols. In a randomized, double-blind, placebo-controlled, crossover study involving 22 healthy volunteers aged 30–60 years, consumption of a black soybean cookie containing flavan-3-ols, cyanidin-3-O-glucoside, and isoflavones for four weeks significantly increased NO_2_/NO_3_ concentration in plasma and urine, contributing to improved vascular function. After consuming the black soybean cookie, a decrease in vascular age was observed in 14 of the 22 participants whose vascular ages were higher than their chronological ages before the trial. Additionally, there was a reduction in systolic and diastolic blood pressure and 8-hydroxy-2’-deoxyguanosine (8-OHdG), a biomarker of oxidative stress (41). The cardioprotective effects of other polyphenols are exemplified in Supplementary Material 2.

In addition to the risk mentioned above factors, evidence has emerged in recent years suggesting a correlation between intestinal microbiota and the occurrence of cardiovascular diseases (42). The interrelation of intestinal microbiota with the heart occurs through metabolites such as bile acids (BAS), short-chain fatty acids (SCFA), trimethylamine N-oxide (TMAO), peptide YY (PYY), and glucagon-like peptide-1 (GLP-1), which are reabsorbed in the intestine and transferred to the circulatory system. SCFA is a beneficial metabolite, while TMAO and endotoxin (LPS) can worsen CVD (43). An imbalance of intestinal microbiota, termed dysbiosis, is generally associated with a reduction in this microbial flora and an increase in pathogenic microorganisms. Dysbiosis is known to occur with aging and the development of age-related diseases such as cardiovascular diseases (44).

The relationship between polyphenol consumption, intestinal microbiota, and cardiovascular diseases has also sparked interest in the scientific community. A cross-sectional study conducted by Li et al. (45) with 200 women aged 52–72 years evaluated the relationship between dietary polyphenol consumption (2,128.9 ± 961.7 mg/day), intestinal microbiome, and the risk of cardiovascular events. A series of metabolites were identified and positively correlated with 34 genera, mainly *Firmicutes* and *Bacteroidetes*. The study concluded that dietary polyphenols may reduce the risk of CVD and modulate the intestinal microbiome (45). Another similar research is described in Supplementary Material 1.

Despite evidence pointing to the beneficial effects of polyphenols on cardiac function, more clinical studies involving the elderly population should be conducted with well-established methodological approaches, with a significant number of volunteers, taking into account the influence of dietary habits, physical activity, and medications on the cardioprotective properties of these compounds and their relationship with the intestinal microbiome and CVD.

### 3.3 Alzheimer, dementia, and other neurodegenerative disorders

Dementia is a progressive and chronic disease with an incidence that increases with age, being rare before the age of 40 (46, 47). Given the lack of effective treatments for dementia, it is crucial to focus on prevention by addressing modifiable and protective risk factors.

The risk of dementia and cognitive decline has been associated with factors compromising cardiovascular function, such as high body mass index, elevated blood glucose, systolic hypertension, and dyslipidemia (47, 48). It has been demonstrated in humans that polyphenolic compounds have a direct effect by crossing the blood-brain barrier accumulating in brain tissue and cerebrospinal fluid (49).

Consumption of plant-derived polyphenols may delay the onset of neurodegenerative diseases and improve the quality of life for those already diagnosed. Some neuroprotective polyphenols may be important for healthy aging as they have been shown to reduce oxidative stress brain atrophy, modulate neuroinflammation and adaptive immunity, and improve cognition, mood, visual functions, language, and verbal memory (49–53). A Mediterranean diet or around one serving per day of leafy green vegetables and foods rich in lutein, α-tocopherol, and kaempferol may help reduce cognitive decline and dementia (54).

### 3.4 Osteoporosis

Osteoporosis (OP) is a chronic age-related systemic skeletal disease characterized by reduced bone mineral density and compromised bone microarchitecture due to an increased osteoclast function that can induce fragility fractures (55). This disease, characteristic of female menopause and aging periods, is also associated with obesity, immune system dysfunction, oxidative stress, and changes in the intestinal microbiota (55, 56). Although women represent most of the population suffering from OP, men can also be affected (57).

The normal metabolic balance between bone formation and degradation, mediated by osteoblasts and osteoclasts, is altered with aging. The decline in estrogen is directly linked to changes in this balance (58). Due to the release of inflammatory cytokines by bone cells (mainly L-1β, IL-6, and TNF-α) caused by estrogen deficiency, there is an increase in osteoclast and a reduction in osteoblast activities (59).

OP treatment involves anabolic and resorptive drugs, lifestyle changes, and supplementation with calcium and vitamin D (55). However, several studies demonstrate that polyphenols can improve bone health in different ways, such as estrogenic activity (60, 61) and antioxidant and anti-inflammatory properties, in addition to gut microbiota modulation (62).

As antioxidants, polyphenols can eliminate reactive oxygen species, inhibiting the effects of oxidative stress on osteogenic processes and modulating signaling pathways that activate osteocytes and osteoblasts (56). Preclinical studies indicate that polyphenols, due to their antioxidant activity, can modulate the differentiation and activity of osteoblasts and osteoclasts through epigenetic regulations, which result in increased deposition and decreased resorption of bones (62).

On the other hand, clinical studies demonstrate that polyphenols such as resveratrol, genistein, daidzein, and equol, a metabolite of daidzein naturally produced by bacteria in the gut of some individuals, improve bone health by increasing body bone mineral density (BMD) (especially of the lumbar spine, femoral colon, and total hip), due to their estrogenic activity. These compounds can also increase osteoblast activity with a concomitant reduction in osteoblast activity (60, 61). Resveratrol is a phytoestrogen capable of modulating estrogenic activity by increasing the gene expression of osteoprotegerin, a protein that inhibits the receptor activator of nuclear factor kappa B ligand (RANKL), neutralizing the differentiation and activity of osteoclasts (63). The effect of polyphenols in some clinical trials can be seen in Supplementary Material 2.

The RESHAW was a trial of 24-month, randomized, double-blind, placebo-controlled, two-period crossover intervention that evaluated the effect of resveratrol supplementation on bone health in 125 postmenopausal, 45–85 years aged women without hormone replacement therapy. After 12 months of resveratrol supplementation (75 mg twice daily - Veri-te^®^ capsules - Evolva SA, Reinach, Switzerland), positive effects were observed on BMD in the lumbar spine and femoral neck. Increasing BMD at the femoral neck improved the T-score and reduced the probability of major hip fractures by 10 years. These observed effects were more pronounced in women with poor bone health biomarker status. Furthermore, there was a 7.24% reduction in plasma of C-terminal telopeptide type-1 collagen (CTX), and no changes in osteocalcin were observed (64).

Evidence suggests that dietary interventions with polyphenol-rich foods or polyphenol-rich extracts contribute positively to the management of OP (65). In the randomized study (12-month, double-blinded, placebo-controlled clinical trial of 78 postmenopausal osteopenic women aged 60–85 years), the effect of red clover extract (RCE), rich in isoflavone aglycones and probiotics was evaluated. The participants were divided into the RCE treatment group (Herrens Mark ApS - 60 mg aglycones/day) and the control group. After an observation period of 12 months, the RCE group presented an attenuated loss of BMD in the lumbar spine, femoral neck, and trochanter and reduced plasma CTX. More than that, the RCE group did not present altered levels of other bone turnover markers. It stimulated equol production in 55% of the treated participants, suggesting that probiotics positively influenced the gut microbiota (66).

### 3.5 Cancer

Aging has been a challenge for contemporary public health, as well as a risk factor for oncogenesis due to functional changes typical of older adults. It is the second leading cause of deaths worldwide, causing more deaths than all coronary heart disease or all strokes. It accounts for 21.7% of non-communicable diseases and may occur in any part of the body (67).

Increased life expectancy raises concerns about promoting healthy aging and quality of living, and nutrition appears to be a good strategy for preventing some age-related diseases. Therefore, understanding the molecular mechanisms behind aging associated with the development of cancers more susceptible to this process is indispensable in developing effective ways to manage the disease (68).

Aging is the primary risk factor for the development of many diseases, including cancer, and this unwelcome relationship likely arises for many reasons, but mostly due to the accumulation of genetic mutations that may be inherited, induced by environmental factors, or result from DNA replication errors and epigenetic alterations (69). However, some cancer types may be reduced by lifestyle changes, such as not smoking, limiting sun exposure, being physically active, and maintaining a healthy diet (67).

Regarding treatment, the conventional options include surgical intervention, systemic therapy and radiotherapy, and taking chemotherapeutic drugs that can be performed alone or in combination (67). However, side effects and toxicity of ones still represent drawbacks to overcome since cancer progression and mortality remain challenging due to these limitations. For this reason, research efforts have been made to find new complementary and alternative approaches that aim to alleviate the suffering of cancer patients (67, 68).

A range of epidemiological and nutritional studies have linked the consumption of fresh fruits and vegetables to a lower risk of cancer incidence and/or improved cancer prognosis after diagnosis. Based on these observations, many preclinical and clinical studies have focused on evaluating the potential of dietary bioactive compounds (e.g., carotenoids, phenolic acids, polyphenols, carbohydrate polymers, and lipids) as antineoplastic agents (70).

Polyphenols, for example, may interfere in the carcinogenesis process of tumor growth and spread, acting on several targets involved in cell proliferation, apoptosis, angiogenesis, and processes involving drug and radiation resistance (71). Also, they may act by reversing epigenetic changes related to DNA methylation and histone modifications and interacting with non-coding (7).

Resveratrol, for example, is a non-flavonoid polyphenol that may modify signaling pathways that affect gene expression, impacting epigenetic mechanisms. Instead, in prostate cancer, it may activate miRNA-mediated regulation of PTEN (phosphatase and tensin homolog) (72). Meanwhile, breast cancer may trigger apoptosis and intracellular Ca^2+^ changes due to DNMT inhibition, followed by increased *ATP2A3* gene expression (73). Hesperetin also may inhibit histone H3K79 methylation by the degradation Disruptor of telomeric silencing 1 (Dot1) expression through CBP-mediated acetylation and, consequently, a reduction in cell migration and invasion in gastric, breast, lung, liver, and colon cancer (74).

Another study demonstrated that kaempferol has been shown to inhibit the proliferation of human glioma cells through inactivation of nuclear factor-kappa B (NF-κB) signaling. It inhibited cell migration and invasion potential, indicating anti-tumor effects (75). Likewise, resveratrol, curcumin, and quercetin combination induced ROS apoptosis in 4T1 breast cancer cells, altering the tumor microenvironment and enhancing the anti-tumor effect (76). Supplementary Materials 1, 2 show additional data reported in the literature concerning the biological effects of dietary polyphenols on cancer.

A body of scientific evidence demonstrates that polyphenols represent a great option for oncological therapy, even complementary, adjuvant, or supplementary alternative, since they may exert regulatory effects in several signaling pathways related to cancer development and progression. However, there are still great challenges to overcome regarding the effectiveness of polyphenols, including bioavailability and stability, as well as metabolic, epigenetic, and gut microbiota interactions.

It is also necessary to improve the understanding of the molecular mechanisms by which polyphenols act as antineoplastic agents, as well as to conduct randomized, double-blind, placebo-controlled clinical trials to evaluate the efficacy, tolerability, and safety of these compounds to establish their potential in terms of appropriate doses, the most effective routes of administration, and for which type of cancer such a therapeutic approach may be most effective.

## 4 A peek into personalized nutrition in the “health aging-context”

The aging process brings with it significant changes to the body. These changes include morphological, psychological, functional, and biochemical alterations^1^. As a result, some illnesses related to nutritional and sensory deficiencies, bone wear, and changes in the hormonal, digestive, and muscular systems, among others, arise. Therefore, managing nutrition in aging requires a personalized approach (77).

Personalized nutrition involves adapting the older person’s diet according to their health conditions, preferences, and nutritional needs. Personalized approaches allow health professionals to (1) assess the specific nutritional needs of each older person; (2) develop diets that strengthen the immune system; (3) manage optimal weight and body composition; (4) improve cognitive function through the consumption of essential nutrients; (5) maintain bone health through a balance of calcium, vitamin D and other minerals. Another crucial point of personalized nutrition is the promotion of emotional wellbeing and improved cognition since eating well is a question of physical health, pleasure, and satisfaction. It also seeks to understand how medicines interact with food to ensure the safety and wellbeing of the elderly individual (77).

Personalized nutrition is often interchangeable with nutritional genomics approaches, whose applications give us insight into how nutrients can “turn on or off” switches in our cells, affecting the expression of various genes and, implicitly, the transcription profiles related to these genes, with direct effects displayed in proteomics and metabolomics (78). The food’s components can also affect and be affected by the intestinal microbiota, which is responsible for producing metabolites that act as allosteric regulators and cofactors of epigenetic processes (79).

**FIGURE 4:**
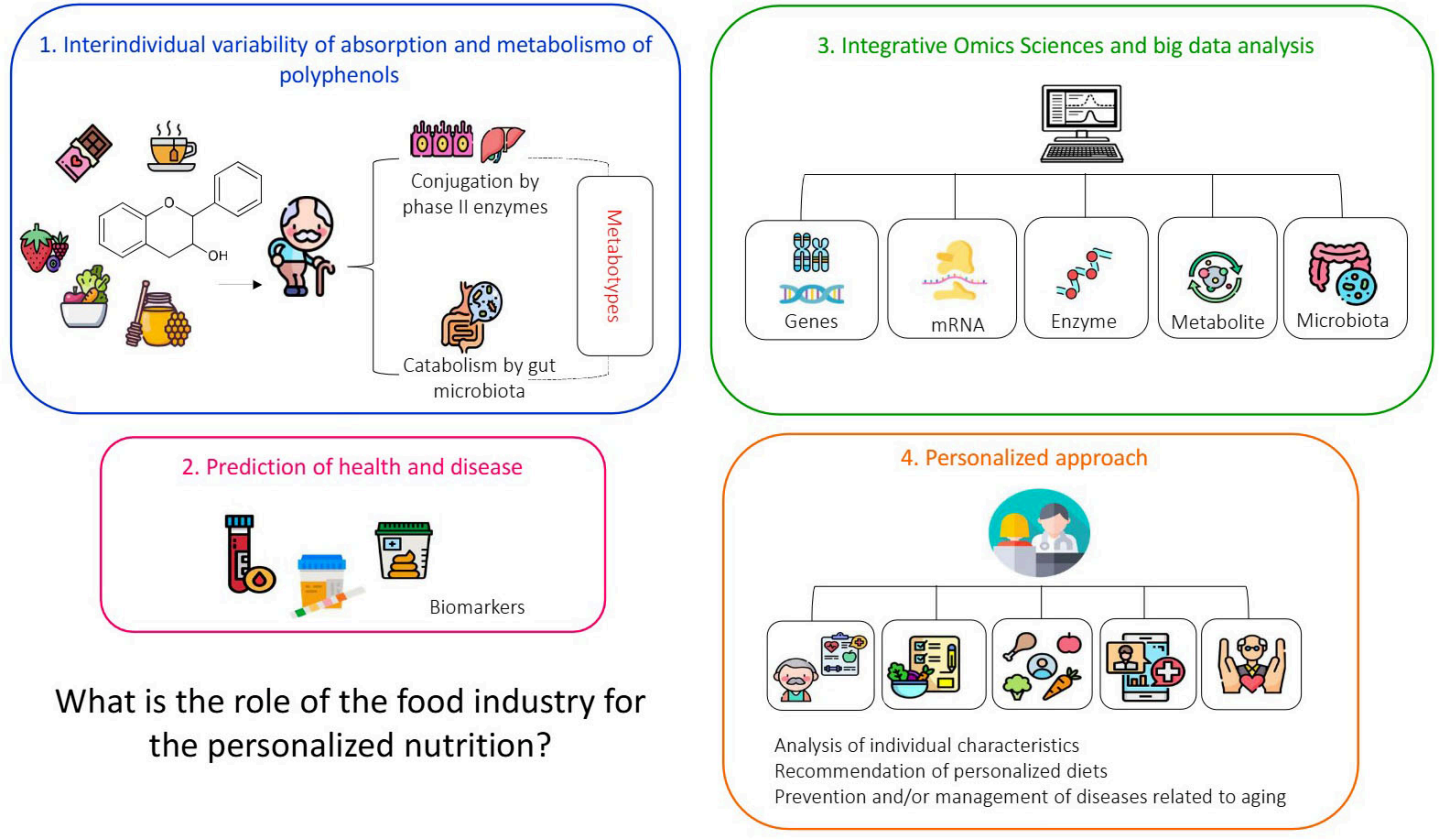
Schematic representation of a service with elements and activities that constitute a personalized nutritional service. Personalized nutrition refers to an individualized approach to designing dietary strategies and plans. This model considers each person’s unique metabolic, physiological, and genetic profile. It requires a comprehensive nutritional assessment, including dietary and body composition analysis, evaluation of family history and biochemical markers, as well as genetic testing and multi-omics approaches (proteomics, transcriptomics, genomics, metabolomics, lipidomics, and epigenomics). Such an integrated evaluation enables a deeper understanding of individual dietary responses, including food intolerances and allergies. These variations, driven by inter-individual differences, influence metabolic pathways and overall homeostasis. Based on this detailed analysis, an optimal diet can then be tailored to meet each person’s specific needs. Source: (7–9).

In clinical practice, targeted nutritional recommendations can begin with a complete nutritional assessment, including dietary history and body assessment, followed by an analysis of family history of disease susceptibility or biochemical parameters, nutrigenetic testing, and use of “omics” tools that also provide helpful information about the individual’s genetic profile, as illustrated in Figure 4 (80).

Technology has the potential to play a crucial role in addressing the challenges of aging and providing a better quality of life and independence. In the nutritional context, technology is a fundamental ally for personalized approaches, using genetic analyses, digital applications, platforms, wearables, IoT, artificial intelligence, and machine learning. Genetic tests help identify potential nutritional deficiencies and predispositions to health conditions, allowing for more effective personalization of eating plans and promoting health in a more targeted manner. Specialized apps allow users to log their meals, track nutrient intake, and receive real-time feedback on their food choices. These apps can provide valuable insights and suggestions for adjustments based on individual goals and needs. For example, if an older adult has diabetes and osteoporosis, an app can help them adapt recipes that include nutrients that strengthen bones while controlling blood sugar levels. Artificial intelligence, machine learning, and deep learning algorithms can also be used to adjust recommendations based on user feedback and changes in their nutritional needs and/or to analyze large volumes of data and identify patterns, trends and correlations as we will discuss.

The “omics” sciences referring to nutri(epi)genomics, metabolomics, and microbiomics may provide a holistic view of how the human body reacts to both nutrients and dietary bioactive compounds while assisting the investigation of individual differences and, thus, favoring personalized nutrition (81).

Nutritional genomics, for example, integrates the so-called nutrigenetics, nutrigenomics, and epigenomics. The science of nutrigenetics analyzes how genetic variability influences how the body metabolizes nutrients. Throughout individualized biomarkers, nutrigenomics seeks to understand how the health-disease process is modified according to the expression of specific genes as a function of nutrients and bioactive compounds from food (81). In turn, epigenetics is defined by changes in the genome, which do not involve changes in the DNA sequence but can result in differences in gene expression. Unlike genetic variations, which are fixed, epigenetic modifications are temporary and may vary within a generation as an immediate response to the environment or metabolism (7, 79).

The field of nutritional genomics gives an insight into how nutrients affect the expression of various genes and, implicitly, the transcription profiles related to those genes, with direct effects displayed in proteomics and metabolomics. It starts from the assumption that nutrients can influence gene expression by acting directly on the genome or indirectly through epigenetics (78). Another aspect to consider is that the components contained in the food we eat can affect and be affected by the intestinal microbiome and that these microorganisms produce metabolites that act as allosteric regulators and cofactors of epigenetic processes (79).

However, there are still significant challenges to be overcome in this area. The first concerns the complexities of food matrices and the fact that most chronic diseases are polygenic, that is, diseases consisting of many genes located at different loci, each with minor effects on the trait, producing quantitative changes (82). The second point is regarding ethical and privacy aspects regarding information security. Therefore, ensuring data is protected and used ethically is crucial to maintaining user trust. Another issue is access, and the high costs of incorporating new technologies for genetic testing, monitoring, and data analysis entail restrictions for developing countries and limiting access to these services for a portion of the population. Finally, the food industry has no clear regulations regarding personalized foods. This can lead to various practices, including, in some cases, questionable practices. Therefore, it is necessary to establish guidelines to ensure the sector’s compliance and quality of services.

### 4.1 Utilization of big data for food science and nutrition: from data collection to action

Multi-omics analyses (i.e., genomics, epigenomics, metabolomics) applied to personalized nutrition (to develop biomarkers of food intake and health status) generate large, complex, and multidimensional datasets. The use of IoT devices further expands this volume of information, producing heterogeneous data in terms of content, structure, and storage formats (83).

Such complexity requires robust methods of curation, integration, and storage, as well as advanced statistical strategies and computational models capable of extracting meaningful patterns (84). In this scenario, big data techniques have become indispensable for the integrated collection, processing, and analysis of these datasets, thereby enabling more precise investigations into organ-system-organisms interactions, as well as more comprehensive assessments of nutrient and bioactive compounds intake, in addition to the effects of drugs or other therapeutic interventions (85).

The concept of big data encompasses a set of methodologies and tools (e.g., data mining, statistics, artificial intelligence (AI), predictive analytics, natural language processing (NLP), etc.) (86) that have been increasingly applied in Food Science, Technology, and Nutrition areas. These approaches centralize the management of large-scale records, allowing the rapid identification of patterns and the prediction of trends based on massive, diverse, and high-speed datasets^2^. Thus, understanding the application of data science may help us locate certain information and, mainly, apply it assertively in choosing nutritional interventions and/or disease prevention/management.

In general terms, big data is characterized by large volumes of structured, semi-structured, and unstructured data generated at high velocity, which therefore require specific technologies for proper analysis. These features are traditionally summarized by the “3Vs”: Volume, Velocity, and Variety, with a fourth “V,” “Veracity,” being particularly relevant in agriculture, nutrition and health, as it reflects the reliability of the data (86). According to The World Health Organization (WHO), big data refers to accumulating, complex, and versatile information that requires storage capacities measures in terabytes (10^12^ bytes), petabytes (10^1^5 bytes), or even zettabytes (10^21^ bytes^3^). However, for a given environment to be considered big data, the sheer volume of information must be accompanied by the speed with which it can be processed to inform timely decision-making (86).

Each of these dimensions presents both challenges and opportunities. Volume and variety allow for broader analyses but demand tools capable of handling diverse formats, ranging from spreadsheets and structured files to media such as audio, images, video, and text (86). These unstructured formats can be particularly useful to assessing eating habits or delivering nutritional monitoring messages. Veracity, in turn, is essential to ensure data quality and trustworthiness, since inaccurate information may compromise nutritional decisions with significant impacts on public health (86).

In practice, predictive algorithms and decision-support systems already enable more individualizes dietary recommendations tailored to lifestyle, metabolic status, and specific goals (e.g., weight loss, muscle gain, cholesterol reduction). The emphasis on data “Veracity,” combined with Volume, Velocity and Variety, thus becomes a prerequisite for safe and effective nutritional interventions (86). For instance, the Stance4Health application, developed by Hinojosa-Nogueira et al. (87), integrates clinical parameters, microbiome data, and information from wearable devices to optimize gut microbiota activity and promote long-term user adherence to healthy dietary strategies (87).

The economic importance of this field is underscored by the growing interest of global corporations such as Nestlé and IBM, which have invested heavily in big data solutions for personalized nutrition and healthcare^4,5^. In 2016 alone, 46% of big data investors in the United States allocated approximately US$3 billion to digital technologies in the agri-food chain, highlighting the transformative potential of this area for food and nutrition innovation (88).

## 5 Impact and importance of technology and personalized nutrition in the food industry

There is no denying that there is a market or niche opportunity for the food industry to develop new food products aimed at senior citizens. However, designing food for this population segment requires specialized efforts since meeting their needs involves awareness of their physiological, psychological, nutritional, and socioeconomic conditions. Healthy food for seniors is expressed as “food that is not good for them” since the aging body cannot tolerate any excess food, and diseases produce limitations in eating. At the same time, there is a preference for practicality when eating, since connected to the modern pace of life, “there is no time to waste.” Therefore, healthy foods that are easy to chew and digest are welcome!

In this sense, nutritional genomics and personalized nutrition fields represent a unique opportunity for innovation in the food industry, enabling the development of personalized products and food solutions that align with the ONU 2030 Agenda^6^. The personalized approach encourages exploring different perspectives regarding producing healthy, tasty, safe, convenient, and affordable foods. In contrast, personalized nutrition integrates an individual’s food preferences and sustainability values into dietary recommendations, guiding them toward environmentally friendly food choices (86).

From this broader perspective, “farm-to-home” applications that integrate “omics” technologies into the food supply chain can improve food processing, food safety and quality assurance, and personalization of food production, distribution, and consumer experience. This includes technologies such as 3D food printing or personalizing purchases for very specific micro-markets with customized delivery options. Designing foods and snacks tailored to meet personalized nutritional recommendations is possible. These products are formulated to support health and wellbeing according to the user’s profile. Or, create menus, dishes, portions, and side dishes that are personalized according to consumer data and preferences. In addition to adding value by using quality ingredients, innovative techniques, and creative presentations,

According to an article published in Faster Capital magazine in June 2024, the food industry for senior nutrition may use technology and innovation to create value and differentiation for its products and services and to improve customer experience and satisfaction. For example, it can develop innovative packaging and sensors to monitor its products’ quality, freshness and shelf life. It can also use artificial intelligence and big data to analyze consumer behavior and preferences and customize and personalize its products and services. For example such as (1) Plant-based foods and alternative proteins that offer high-quality protein, fiber, and micronutrients while reducing the intake of saturated fat, cholesterol and antibiotics; (2) Functional foods and drinks that improve immunity, cognition, digestion, bone and intestinal health; (3) Ready-to-drink rich in proteins to prevent muscle loss and malnutrition; (4) Nutritionally balanced foods, with controlled portions and frozen for your convenience, and so on^7^).

However, there is a fundamental barrier to the production of personalized foods. Today, the agri-food system focuses on mass production to deliver food products at high productivity rates and economies of scale. The idea is offering convenience with a longer shelf life, often ignoring regional context and cultural tradition, and this is practically the opposite of the ideal system for delivering personalized foods.

This means that to meet future demands aligned with nutritional genomics and, consequently, personalized nutrition, it will be necessary for the production chain to seek alternative ways to the obsolescence of traditional agri-food systems to improve how food is grown, processed, and consumed, which will result in the production of food with a chemical composition more suited to consumer needs (84, 89).

Process modifications and new food technologies are proposed as potentially holding the key to the necessary transformation toward mass customization and personalization of new food products. However, the extent to which this can be achieved and the costs of delivering personalized products are currently unclear.

For now, it is possible to glimpse the development of beverages and foods as preventive agents or for the treatment of individuals, families, or subgroups predisposed to a specific disease, similar to ketogenic diets indicated for the treatment of patients with intractable epilepsy or diets balanced in fatty acids for patients with chronic inflammatory diseases such as arthritis, asthma, ulcerative colitis, lupus, etc., as well as in patients with coronary artery disease and hypertension.

In the coming years, the concept of personalized diets is expected to expand knowledge and understanding of gene-diet interactions, in addition to serving as a basis for the development of personalized ready-to-eat meals and various products that offer consumers the ease of integrating personalized nutrition consulting services into their daily diet and meal routines, as this approach would allow extended adherence by consumers concerning lifestyle and eating habits, thus providing more effective results in maintaining health and wellbeing.

## 6 Conclusion and future perspectives

Food is much more than a way for the body to obtain the nutrients it needs to survive. To talk about good nutrition is to talk about harmony. Eating well means being emotionally well and ready for an active life. A well-nourished body meets a healthy mind. A proper diet contributes to the absence of diseases and leads to complete physical, mental, and social wellbeing.

This is a highly complex topic related to health, encompassing multiple areas of knowledge. It is important to recognize that the effects of diet and nutritional status on health are multifaceted. Consequently, analyzing these effects requires technical expertise to interpret population-level data, particularly as health promotion has increasingly been emphasized as a strategy in both public health and food production in recent years.

So, we are left with the question: What can we expect from nutritional genomics and personalized nutrition in the future? And what is the role of the food industry in this endeavor?

Technology is a field in constant and rapid development, and both biological and nutritional sciences have benefited from this development and progressed simultaneously. “Omics” sciences in the personalized context can provide a holistic view of how the human body reacts to both nutrients and bioactive compounds in the diet while also helping to investigate individual differences and thus favoring individualized diets for older people, in addition to offering new possibilities for innovation in the food industry production chain that aim to improve not only the nutritional characteristics of foods but also improvements in food processing, food safety, and quality assurance.

These are just a few examples of the possibilities that these new fields of knowledge can offer. They will yield short- and long-term benefits for human health, particularly for older people with chronic diseases, revealing new nutrient-gene interactions, metabolic processes, and nutritional requirements, helping to fill the gaps between comprehensive public health messages and individualized dietary guidelines. However, an evidence-based approach is required to validate that personalized recommendations are effective health benefits for individuals and do not cause harm.

## Footnotes

1
https://www.who.int/news-room/fact-sheets/detail/healthy-diet

2
https://www.ibm.com/analytics/big-data-analytics

3
https://www.who.int/europe/news/item/26-05-2021-using-big-data-to-inform-health-care-opportunities-challenges-and-considerations

4
https://www.thecasecentre.org/products/view?id=157889

5
https://www.ibm.com/watson-health

6
https://sdgs.un.org/goals

7
https://fastercapital.com/content/Geriatric-nutrition-startup-Revolutionizing-Geriatric-Nutrition–A-Startup-s-Journey.html

## References

[1] Whitley E Benzeval M Popham F. Population priorities for successful aging: a randomized vignette experiment. *J Gerontol Ser B.* (2018) 75:293–302. 10.1093/geronb/gby060 29878183 PMC6974399

[2] United Nations. *Department of Economic and Social Affairs PD (2019). WPA 2019: H (ST/ESA/SER. A. World Population Ageing 2019: Highlights.* New York, NY: United Nations (2019). p. 1–40.

[3] Jha A Sit N. Extraction of bioactive compounds from plant materials using combination of various novel methods: a review. *Trends Food Sci Technol.* (2022) 119:579–91. 10.1016/J.TIFS.2021.11.019

[4] Hossain M Wazed M Asha S Amin M Shimul I. Dietary phytochemicals in health and disease: mechanisms, clinical evidence, and applications–a comprehensive review. *Food Sci Nutr.* (2025) 13:101. 10.1002/fsn3.70101 40115248 PMC11922683

[5] Guglielmetti S Bernardi S Del Bo’ C Cherubini A Porrini M Gargari G Effect of a polyphenol-rich dietary pattern on intestinal permeability and gut and blood microbiomics in older subjects: study protocol of the MaPLE randomised controlled trial. *BMC Geriatr.* (2020) 20:77. 10.1186/s12877-020-1472-9 32102662 PMC7045478

[6] WHO. *World Report on Ageing and HeAltH.* Geneva: WHO (2015).

[7] Borsoi F Neri-Numa I de Oliveira W de Araújo F Pastore G. Dietary polyphenols and their relationship to the modulation of non-communicable chronic diseases and epigenetic mechanisms: a mini-review. *Food Chem Mol Sci.* (2023) 6:100155. 10.1016/j.fochms.2022.100155 36582744 PMC9793217

[8] Quideau S Deffieux D Douat-Casassus C Pouységu L. Plant polyphenols: chemical properties, biological activities, and synthesis. *Angew Chem Int.* (2011) 50:586–621. 10.1002/anie.201000044 21226137

[9] Sharma R Padwad Y. Perspectives of the potential implications of polyphenols in influencing the interrelationship between oxi-inflammatory stress, cellular senescence and immunosenescence during aging. *Trends Food Sci Technol.* (2020) 98:41–52. 10.1016/j.tifs.2020.02.004

[10] de Araújo F de Paulo Farias D Neri-Numa I Pastore G. Polyphenols and their applications: an approach in food chemistry and innovation potential. *Food Chem.* (2021) 338:7535. 10.1016/J.FOODCHEM.2020.127535 32798817

[11] Neri-Numa I Cazarin C Ruiz A Paulino B Molina G Pastore G. Targeting flavonoids on modulation of metabolic syndrome. *J Funct Foods.* (2020) 73:104132. 10.1016/j.jff.2020.104132

[12] Gadecka A Bielak-Zmijewska A Gadecka A Bielak-Zmijewska A. Slowing down ageing: the role of nutrients and microbiota in modulation of the epigenome. *Nutrients.* (2019) 11:1251. 10.3390/nu11061251 31159371 PMC6628342

[13] Aruoma O Hausman-Cohen S Pizano J Schmidt M Minich D Joffe Y Personalized nutrition: translating the science of nutrigenomics into practice: proceedings from the 2018 American college of nutrition meeting. *J Am Coll Nutr.* (2019) 38:287–301. 10.1080/07315724.2019.1582980 31099726

[14] Skinner M Lumey L Fleming T Sapienza C Hoyo C Aronica L RW-2018–research workshop: the effect of nutrition on epigenetic status, growth, and health. *J Parenter Enter Nutr.* (2019) 43:627–37. 10.1002/jpen.1536 30997688 PMC6625918

[15] Deng F Li Y Zhao J. The gut microbiome of healthy long-living people. *Aging.* (2019) 11:289–90. 10.18632/aging.101771 30648974 PMC6366966

[16] Lin S Wang Z Lam K Zeng S Tan B Hu J. Role of intestinal microecology in the regulation of energy metabolism by dietary polyphenols and their metabolites. *Food Nutr Res.* (2019) 14:63. 10.29219/fnr.v63.1518 30814920 PMC6385797

[17] Tzounis X Rodriguez-Mateos A Vulevic J Gibson G Kwik-Uribe C Spencer J. Prebiotic evaluation of cocoa-derived flavanols in healthy humans by using a randomized, controlled, double-blind, crossover intervention study. *Am J Clin Nutr.* (2011) 93:62–72. 10.3945/ajcn.110.000075 21068351

[18] Cho S Kim J Lee J Sim J Cho D Bae I Modulation of gut microbiota and delayed immunosenescence as a result of syringaresinol consumption in middle-aged mice. *Sci Rep.* (2016) 6:39026. 10.1038/srep39026 27976725 PMC5157019

[19] Xiao Y Dong J Yin Z Wu Q Zhou Y Zhou X. Procyanidin B2 protects against d-galactose-induced mimetic aging in mice: metabolites and microbiome analysis. *Food Chem Toxicol.* (2018) 119:141–9. 10.1016/J.FCT.2018.05.017 29751077

[20] Philip N Walsh L Philip N Walsh L. Cranberry polyphenols: natural weapons against dental caries. *Dent J.* (2019) 7:20. 10.3390/dj7010020 30823634 PMC6473364

[21] Carmona-Gutierrez D Zimmermann A Kainz K Pietrocola F Chen G Maglioni S The flavonoid 4,4′-dimethoxychalcone promotes autophagy-dependent longevity across species. *Nat Commun.* (2019) 10:651. 10.1038/s41467-019-08555-w 30783116 PMC6381180

[22] Galicia-Garcia U Benito-Vicente A Jebari S Larrea-Sebal A Siddiqi H Uribe K Pathophysiology of type 2 diabetes mellitus. *Int J Mol Sci.* (2020) 21:6275. 10.3390/ijms21176275 32872570 PMC7503727

[23] Palmer A Gustafson B Kirkland J Smith U. Cellular senescence: at the nexus between ageing and diabetes. *Diabetologia.* (2019) 62:1835–41. 10.1007/s00125-019-4934-x 31451866 PMC6731336

[24] Zampieri M Karpach K Salerno G Raguzzini A Barchetta I Cimini F PAR level mediates the link between ROS and inflammatory response in patients with type 2 diabetes mellitus. *Redox Biol.* (2024) 75:103243. 10.1016/j.redox.2024.103243 38906011 PMC11253151

[25] Brownlee M. Biochemistry and molecular cell biology of diabetic complications. *Nature.* (2001) 414:813–20. 10.1038/414813a 11742414

[26] Horváth E Magenheim R Kugler E Vácz G Szigethy A Lévárdi F Nitrative stress and poly(ADP-ribose) polymerase activation in healthy and gestational diabetic pregnancies. *Diabetologia.* (2009) 52:1935–43. 10.1007/s00125-009-1435-3 19597800

[27] Giorgi A Tempera I Napoletani G Drovandi D Potestà C Martire S Poly(ADP-ribosylated) proteins in mononuclear cells from patients with type 2 diabetes identified by proteomic studies. *Acta Diabetol.* (2017) 54:833–42. 10.1007/s00592-017-1013-y 28608282

[28] Zampieri M Bacalini M Barchetta I Scalea S Cimini F Bertoccini L Increased PARylation impacts the DNA methylation process in type 2 diabetes mellitus. *Clin Epigenet.* (2021) 13:114. 10.1186/s13148-021-01099-1 34001206 PMC8130175

[29] Kim Y Keogh J Clifton P. Polyphenols and glycemic control. *Nutrients.* (2016) 8:17. 10.3390/nu8010017 26742071 PMC4728631

[30] García-Martínez B Ruiz-Ramos M Pedraza-Chaverri J Santiago-Osorio E Mendoza-Núñez V. Effect of resveratrol on markers of oxidative stress and sirtuin 1 in elderly adults with type 2 diabetes. *Int J Mol Sci.* (2023) 24:7422. 10.3390/ijms24087422 37108584 PMC10138491

[31] Mahjabeen W Khan D Mirza S. Role of resveratrol supplementation in regulation of glucose hemostasis, inflammation and oxidative stress in patients with diabetes mellitus type 2: a randomized, placebo-controlled trial. *Complement Ther Med.* (2022) 66:102819. 10.1016/j.ctim.2022.102819 35240291

[32] Hashimoto N Tanaka T. Role of miRNAs in the pathogenesis and susceptibility of diabetes mellitus. *J Hum Genet.* (2017) 62:141–50. 10.1038/jhg.2016.150 27928162

[33] Gaziano T. *“Cardiovascular Diseases Worldwide” Public Health Approach to Cardiovascular Disease Prevention & Management.* Boca Raton, FL: CRC Press (2022). p. 8–18. 10.1201/b23266-2

[34] Liberale L Badimon L Montecucco F Lüscher T Libby P Camici G. Inflammation, aging, and cardiovascular disease. *J Am Coll Cardiol.* (2022) 79:837–47. 10.1016/j.jacc.2021.12.017 35210039 PMC8881676

[35] Fiordelisi A Iaccarino G Morisco C Coscioni E Sorriento D. NFkappaB is a key player in the crosstalk between inflammation and cardiovascular diseases. *Int J Mol Sci.* (2019) 20:1599. 10.3390/ijms20071599 30935055 PMC6480579

[36] Gal R Praksch D Kenyeres P Rabai M Toth K Halmosi R Hemorheological alterations in patients with heart failure with reduced ejection fraction treated by resveratrol. *Cardiovasc Ther.* (2020) 2020:7262474. 10.1155/2020/7262474 32695229 PMC7350166

[37] Iqbal I Wilairatana P Saqib F Nasir B Wahid M Latif M Plant polyphenols and their potential benefits on cardiovascular health: a review. *Molecules.* (2023) 28:6403. 10.3390/molecules28176403 37687232 PMC10490098

[38] Trindade L da Silva D Baião D Paschoalin VMF. Increasing the power of polyphenols through nanoencapsulation for adjuvant therapy against cardiovascular diseases. *Molecules.* (2021) 26:4621. 10.3390/molecules26154621 34361774 PMC8347607

[39] Constantinescu T Mihis A. Resveratrol as a privileged molecule with antioxidant activity. *Food Chem Adv.* (2023) 3:100539. 10.1016/j.focha.2023.100539

[40] Zhang L Chen J Yan L He Q Xie H Chen M. Resveratrol ameliorates cardiac remodeling in a murine model of heart failure with preserved ejection fraction. *Front Pharmacol.* (2021) 12:646240. 10.3389/fphar.2021.646240 34177571 PMC8225267

[41] Yamashita Y Nakamura A Nanba F Saito S Toda T Nakagawa J Black soybean improves vascular function and blood pressure: a randomized, placebo controlled, crossover trial in humans. *Nutrients.* (2020) 12:2755. 10.3390/nu12092755 32927677 PMC7551904

[42] Wang L Wang S Zhang Q He C Fu C Wei Q. The role of the gut microbiota in health and cardiovascular diseases. *Mol Biomed.* (2022) 3:30. 10.1186/s43556-022-00091-2 36219347 PMC9554112

[43] Jansen V Gerdes V Middeldorp S van Mens T. Gut microbiota and their metabolites in cardiovascular disease. *Best Pract Res Clin Endocrinol Metab.* (2021) 35:101492. 10.1016/j.beem.2021.101492 33642219

[44] Kawamoto S Hara E. Crosstalk between gut microbiota and cellular senescence: a vicious cycle leading to aging gut. *Trends Cell Biol.* (2024) 34:626–35. 10.1016/j.tcb.2023.12.004 38220548

[45] Li Y Xu Y Le Roy C Hu J Steves C Bell J Interplay between the (Poly)phenol metabolome, gut microbiome, and cardiovascular health in women: a cross-sectional study from the TwinsUK cohort. *Nutrients.* (2023) 15:1900. 10.3390/nu15081900 37111123 PMC10141398

[46] Vieira R. Epidemiology of early-onset dementia: a review of the literature. *Clin Pract Epidemiol Ment Heal.* (2013) 9:88–95. 10.2174/1745017901309010088 23878613 PMC3715758

[47] Nichols E Steinmetz J Vollset S Fukutaki K Chalek J Abd-Allah F Estimation of the global prevalence of dementia in 2019 and forecasted prevalence in 2050: an analysis for the global burden of disease study 2019. *Lancet Public Heal.* (2022) 7:e105–25. 10.1016/S2468-2667(21)00249-8 34998485 PMC8810394

[48] Zhang R Beyer F Lampe L Luck T Riedel-Heller S Loeffler M White matter microstructural variability mediates the relation between obesity and cognition in healthy adults. *Neuroimage.* (2018) 172:239–49. 10.1016/j.neuroimage.2018.01.028 29378320

[49] Grabska-Kobyłecka I Szpakowski P Król A Książek-Winiarek D Kobyłecki A Głąbiński A Polyphenols and their impact on the prevention of neurodegenerative diseases and development. *Nutrients.* (2023) 15:3454. 10.3390/nu15153454 37571391 PMC10420887

[50] Siddarth P Li Z Miller K Ercoli L Merril D Henning S Randomized placebo-controlled study of the memory effects of pomegranate juice in middle-aged and older adults. *Am J Clin Nutr.* (2020) 111:170–7. 10.1093/ajcn/nqz241 31711104

[51] Moussa C Hebron M Huang X Ahn J Rissman R Aisen P Resveratrol regulates neuro-inflammation and induces adaptive immunity in Alzheimer’s disease. *J Neuroinflamm.* (2017) 14:1–10. 10.1186/s12974-016-0779-0 28086917 PMC5234138

[52] Zahedi H Hosseinzadeh-Attar MJ Shadnoush M Sahebkar A Barkhidarian B Sadeghi O Effects of curcuminoids on inflammatory and oxidative stress biomarkers and clinical outcomes in critically ill patients: a randomized double-blind placebo-controlled trial. *Phyther Res.* (2021) 35:4605–15. 10.1002/ptr.7179 34080237

[53] Kaplan A Zelicha H Yaskolka Meir A Rinott E Tsaban G Levakov G The effect of a high-polyphenol mediterranean diet (Green-MED) combined with physical activity on age-related brain atrophy: the dietary intervention randomized controlled trial polyphenols unprocessed study (DIRECT PLUS). *Am J Clin Nutr.* (2022) 115:1270–81. 10.1093/ajcn/nqac001 35021194 PMC9071484

[54] Morris M Wang Y Barnes L Bennett D Dawson-Hughes B Booth S. Nutrients and bioactives in green leafy vegetables and cognitive decline. *Neurology.* (2018) 90:4815. 10.1212/WNL.0000000000004815 29263222 PMC5772164

[55] Lisco G Triggiani D Giagulli V De Pergola G Guastamacchia E Piazzolla G Endocrine, metabolic, and immune pathogenesis of postmenopausal osteoporosis. Is there a therapeutic role in natural products? *Endocr Metab Immune Disord Drug Targets.* (2023) 23:1278–90. 10.2174/1871530323666230330121301 37005529

[56] Marcucci G Domazetovic V Nediani C Ruzzolini J Favre C Brandi M. Oxidative stress and natural antioxidants in osteoporosis: novel preventive and therapeutic approaches. *Antioxidants.* (2023) 12:373. 10.3390/antiox12020373 36829932 PMC9952369

[57] Morin S Feldman S Funnell L Giangregorio L Kim S McDonald-Blumer H Clinical practice guideline for management of osteoporosis and fracture prevention in Canada: 2023 update. *Can Med Assoc J.* (2023) 195:E1333–48. 10.1503/cmaj.221647 37816527 PMC10610956

[58] Khosla S. Pathogenesis of age-related bone loss in humans. *J Gerontol Ser A Biol Sci Med Sci.* (2013) 68:1226–35. 10.1093/gerona/gls163 22923429 PMC3826857

[59] Damani J De Souza M VanEvery H Strock N Rogers C. The role of prunes in modulating inflammatory pathways to improve bone health in postmenopausal women. *Adv Nutr.* (2022) 13:1476–92. 10.1093/advances/nmab162 34978320 PMC9526830

[60] Corbi G Nobile V Conti V Cannavo A Sorrenti V Medoro A Equol and resveratrol improve bone turnover biomarkers in postmenopausal women: a clinical trial. *Int J Mol Sci.* (2023) 24:12063. 10.3390/ijms241512063 37569440 PMC10419295

[61] Squadrito F Imbalzano E Rottura M Arcoraci V Pallio G Catalano A Effects of genistein aglycone in glucocorticoid induced osteoporosis: a randomized clinical trial in comparison with alendronate. *Biomed Pharmacother.* (2023) 163:114821. 10.1016/j.biopha.2023.114821 37167726

[62] Bellavia D Caradonna F Dimarco E Costa V Carina V De Luca A Non-flavonoid polyphenols in osteoporosis: preclinical evidence. *Trends Endocrinol Metab.* (2021) 32:515–29. 10.1016/j.tem.2021.03.008 33895073

[63] Zhao H Li X Li N Liu T Liu J Li Z Long-term resveratrol treatment prevents ovariectomy-induced osteopenia in rats without hyperplastic effects on the uterus. *Br J Nutr.* (2014) 111:836–46. 10.1017/S0007114513003115 24073920

[64] Wong R Thaung Zaw J Xian C Howe P. Regular supplementation with resveratrol improves bone mineral density in postmenopausal women: a randomized, placebo-controlled trial. *J Bone Miner Res.* (2020) 35:2121–31. 10.1002/jbmr.4115 32564438 PMC7689937

[65] George K Munoz J Ormsbee L Akhavan N Foley E Siebert S The short-term effect of prunes in improving bone in men. *Nutrients.* (2022) 14:276. 10.3390/nu14020276 35057457 PMC8779167

[66] Lambert M Thybo C Lykkeboe S Rasmussen L Frette X Christensen L Combined bioavailable isoflavones and probiotics improve bone status and estrogen metabolism in postmenopausal osteopenic women: a randomized controlled trial. *Am J Clin Nutr.* (2017) 106:909–20. 10.3945/ajcn.117.153353 28768651

[67] NIH. *What Is Cancer? National Cancer Institute.* Bethesda, MD: NIH (2021).

[68] Arruda H Neri-Numa I Kido L Maróstica Júnior M Pastore G. Recent advances and possibilities for the use of plant phenolic compounds to manage ageing-related diseases. *J Funct Foods.* (2020) 75:104203. 10.1016/j.jff.2020.104203

[69] Pelissari F Neri-Numa I Molina G Ferreira D Pastore G. Potential of nanoparticles as drug delivery system for cancer treatment. In: <snm>Inamuddin, Asiri A</gnm>, <snm>Mohammad A</gnm> editors. *Applications of Nanocomposite Materials in Drug Delivery.* Amsterdam: Elsevier (2018). p. 431–68. 10.1016/B978-0-12-813741-3.00019-4

[70] Wang X Chan Y Wong K Yoshitake R Sadava D Synold T Mechanism-driven and clinically focused development of botanical foods as multitarget anticancer medicine: collective perspectives and insights from preclinical studies, IND applications and early-phase clinical trials. *Cancers.* (2023) 15:701. 10.3390/cancers15030701 36765659 PMC9913787

[71] Moar K Yadav S Pant A Deepika, Maurya PK. Anti-tumor effects of polyphenols via targeting cancer driving signaling pathways: a review. *Indian J Clin Biochem.* (2024) 39:470–88. 10.1007/s12291-024-01222-y 39346722 PMC11436542

[72] Zhong Z Guo X Zheng Y. Network pharmacology-based and molecular docking analysis of resveratrol’s pharmacological effects on type i endometrial cancer. *Anticancer Agents Med Chem.* (2022) 22:1933–44. 10.2174/1871520621666211015140455 34773964 PMC9241081

[73] Izquierdo-Torres E Hernández-Oliveras A Meneses-Morales I Rodríguez G Fuentes-García G Zarain-Herzberg Á. Resveratrol up-regulates ATP2A3 gene expression in breast cancer cell lines through epigenetic mechanisms. *Int J Biochem Cell Biol.* (2019) 113:37–47. 10.1016/j.biocel.2019.05.020 31173924

[74] Wang S Sheng H Zheng F Zhang F. Hesperetin promotes DOT1L degradation and reduces histone H3K79 methylation to inhibit gastric cancer metastasis. *Phytomedicine.* (2021) 84:153499. 10.1016/J.PHYMED.2021.153499 33667841

[75] dos Santos J Suzan A Bonafé G Fernandes A Longato GB Antônio MA Kaempferol and biomodified kaempferol from sophora japonica extract as potential sources of anti-cancer polyphenolics against high grade glioma cell lines. *Int J Mol Sci.* (2023) 24:10716. 10.3390/ijms241310716 37445894 PMC10341967

[76] Li C Xu Y Zhang J Zhang Y He W Ju J The effect of resveratrol, curcumin and quercetin combination on immuno-suppression of tumor microenvironment for breast tumor-bearing mice. *Sci Rep.* (2023) 13:13278. 10.1038/s41598-023-39279-z 37587146 PMC10432483

[77] Longo V Anderson R. Nutrition, longevity and disease: from molecular mechanisms to interventions. *Cell.* (2022) 185:1455–70. 10.1016/J.CELL.2022.04.002 35487190 PMC9089818

[78] Singh V. Current challenges and future implications of exploiting the ‘OMICS’ data into nutrigenetics and nutrigenomics for personalized diagnosis and nutrition-based care. *Nutrition.* (2023) 110:112002. 10.1016/J.NUT.2023.112002 36940623

[79] Neri Numa I Pastore G. Novel insights into prebiotic properties on human health: a review. *Food Res Int.* (2020) 131:108973. 10.1016/j.foodres.2019.108973 32247494

[80] Camp K Trujillo E. Position of the academy of nutrition and dietetics: nutritional genomics. *J Acad Nutr Diet.* (2014) 114:299–312. 10.1016/j.jand.2013.12.001 24439821

[81] Braconi D Cicaloni V Spiga O Santucci A. Personalized nutrition and omics technologies: current status and perspectives. *Food Technol Disrupt.* (2021) 52:37–71. 10.1016/B978-0-12-821470-1.00007-0

[82] Pointner A Haslberger A. Personalized nutrition for healthy aging, a review. *Adv Precis Nutr Pers Heal Aging.* (2022) 85:97–143. 10.1007/978-3-031-10153-3_5

[83] Dikarlo P Dorst I Moskalenko O Yateem M. Precision nutrition from the view of the gut microbiome. In: Haslberger AG editor. *Advances in Precision Nutrition, Personalization and Healthy Aging.* Cham: Springer (2022). p. 67–96. 10.1007/978-3-031-10153-3_4

[84] Bakhtin P Khabirova E Kuzminov I Thurner T. The future of food production – a text-mining approach. *Technol Anal Strateg Manag.* (2020) 32:516–28. 10.1080/09537325.2019.1674802

[85] Alizadeh M Sampaio Moura N Schledwitz A Patil S Ravel J Raufman J. Big data in gastroenterology research. *Int J Mol Sci.* (2023) 24:2458. 10.3390/IJMS24032458 36768780 PMC9916510

[86] Numa I Wolf K Pastore G. FoodTech startups: technological solutions to achieve SDGs. *Food Hum.* (2023) 1:358–69. 10.1016/j.foohum.2023.06.011

[87] Hinojosa-Nogueira D Ortiz-Viso B Navajas-Porras B Pérez-Burillo S González-Vigil V de la Cueva SP Stance4Health nutritional APP: a path to personalized smart nutrition. *Nutrients.* (2023) 15:276. 10.3390/nu15020276 36678148 PMC9864275

[88] Kuzminov I Bakhtin P Khabirova E Kotsemir M Lavrynenko A. *Mapping the Radical Innovations in Food Industry: A Text Mining Study. SSRN Electron Journal.* Moscow: National Research University Higher School of Economics (2018). 10.2139/ssrn.3143721

[89] Desana A. *FoodTech: A New Solution to Make the Food Sector More Sustainable by Combining Tradition and Innovation.* Venice: Università Ca′Foscari Venezia (2021).

