# Supplementary material for: Polyphenols, aging, and health: What can we expect from the food industry in the technology era?
Source: Front Med (Lausanne). 2025 Nov 6;12:1671886. doi: 10.3389/fmed.2025.1671886 (PMC12630120; doi:10.3389/fmed.2025.1671886)
Supplement: Supplementary file 1 [file Table_1.docx]

**Supplementary material 1**. Preclinical studies involving phenolic compounds and their metabolites on the gut microbiota modulation and/or epigenetic regulation.

| **Compound** | **Effect on gut microbiota** | **Major phenolic metabolites** | **Epigenetic effects of metabolites** | **References** |
| --- | --- | --- | --- | --- |
| Ellagic acid and anthocyanins | ↑*Faecalibacterium prausnitzii, Lactobacillus,* and *Eubacterium rectale*  ↓Desulfovibrio sp. and Enterococcus spp. | Not determined | Change the methylation status  ↑SFRP2 expression  ↓DNMT31, DNMT3B and p-STAT3 regulation | (1) |
| Urolithin B | ↑*Akkermansia* | Not determined | ↑ Insulin sensitivity and insulin tolerance  ↓Glucose-induced hyperinsulinemia and intestinal triglyceride content  ↓Inflammation and oxidative stress. | (2) |
| Anthocyanin in mulberry juice:  2-hydroxy-3-(4-hydroxyphenyl) propanoic acid | ↑*Bifidobacteriaceae,* *Lactobacillaceae* and SCFAs | Not determined | ↓oxidative stress level  ↑ SIRT1 and BDNF  Improved the integrity of hippocampal tissue | (3) |
| Ellagic acid | ↑Proprionate-producing bacteria like *Alloprevotell asp.* | Propionic acid | ↓autoimmune encephalomyelitis (EAE) and HDAC activity  ↑Acetylation and inflammatory cytokines | (4) |
|  |  |  |  |  |
| Isoflavones | Stimulated the growth of *B. bifidum DNG6*, *L. lactisSt66*, *L. plantarum 10.960* and *L. rhamnose* and inhibited pathogenic bacteria (*E. coli* and *S. aureus*) *in vitro*. | *O*-desmethylangolensin and equol | **Equol**: ↓ methylation of the cytosine phosphate guanine (CpG) islands in the *BRCA*1 and *BRCA2* promoters in MCF-7 and MDA-MB-231 cells and ↑ *BRCA1* and BRCA2 proteins expression in nuclei and cytoplasm in cell lines MCF-7, MDA-MB-231 and MCF-10a by immunohistochemistry. | Chen et al. (2022)  Bosviel et al. (2012) |
| Quercetin | ↓Verrocomicrobia and ↑ microbiome diversity and abundance of *Actinobacteria*, *Cyanobacteria* and *Firmicutes*. | Acids and aldehydes phenolic | **Chlorogenic acid**: ↓ proliferation, colony formation, invasion, and metastasis of HepG2 cells both *in vitro* and *in vivo* by down-regulating DNMT1 protein expression, ↑ p53 and p21 activity, ↓ cell proliferation and metastasis, in addition inactivated ERK1/2 and reduced *MMP-2* and *MMP-*9 expression in HepG2 cells. | (7)  Y. Liu et al. (2020) |

**Symbol:** ↓ sample induced significant reduction; ↑ sample induced significant increasing. - Not found. **ALP**, alkaline phosphatase; ***BDNF,*** Brain-derived neurotrophic factor; ***BRCA1***, Breast cancer gene 1; ***BRCA2***, Breast cancer gene 2; **cAMP,** cyclic AMP; **ERK,** extracellular signal-regulated protein kinase; **gp91*^phox^***, NADPH oxidase 2; **MMP2,** matrix metalloproteinase-2; MMP9, Matrix metallopeptidase 9; **p21,** cyclin-dependent kinase inhibitor 1; **p22*^phox^***, human neutrophil cytochrome b light chain; **p53,** tumor protein P53; **PKA,** protein kinase A, ***SIRT1,*** Sirtuin 1.

**Reference**

1. Chen L, Jiang B, Zhong C, Guo J, Zhang L, Mu T, et al. Chemoprevention of colorectal cancer by black raspberry anthocyanins involved the modulation of gut microbiota and SFRP2 demethylation. Carcinogenesis [Internet]. 2018 Mar 8;39(3):471–81. Available from: https://academic.oup.com/carcin/article/39/3/471/4817460

2. Chen P, Wang R, Lei J, Feng L, Zhou B. Urolithin B protects mice from diet-induced obesity, insulin resistance, and intestinal inflammation by regulating gut microbiota composition. Food Funct [Internet]. 2024;15(14):7518–33. Available from: https://xlink.rsc.org/?DOI=D4FO02545H

3. Li M, Xu X, Jia Y, Yuan Y, Na G, Zhu L, et al. Transformation of mulberry polyphenols by Lactobacillus plantarum SC-5: Increasing phenolic acids and enhancement of anti-aging effect. Food Res Int [Internet]. 2024 Sep;192:114778. Available from: https://linkinghub.elsevier.com/retrieve/pii/S0963996924008482

4. Han B, Shi L, Bao M-Y, Yu F-L, Zhang Y, Lu X-Y, et al. Dietary ellagic acid therapy for CNS autoimmunity: Targeting on Alloprevotella rava and propionate metabolism. Microbiome [Internet]. 2024 Jun 24;12(1):114. Available from: https://microbiomejournal.biomedcentral.com/articles/10.1186/s40168-024-01819-8

5. Chen P, Sun J, Liang Z, Xu H, Du P, Li A, et al. The bioavailability of soy isoflavones in vitro and their effects on gut microbiota in the simulator of the human intestinal microbial ecosystem. Food Res Int. 2022 Feb;152:110868.

6. Bosviel R, Durif J, Déchelotte P, Bignon YJ, Bernard-Gallon D. Epigenetic modulation of BRCA1 and BRCA2 gene expression by equol in breast cancer cell lines. Br J Nutr. 2012 Oct;108(7):1187–93.

7. Nie J, Zhang L, Zhao G, Du X. Quercetin reduces atherosclerotic lesions by altering the gut microbiota and reducing atherogenic lipid metabolites. J Appl Microbiol. 2019 Dec;127(6):1824–34.

8. Liu Y, Feng Y, Li Y, Hu Y, Zhang Q, Huang Y, et al. Chlorogenic Acid Decreases Malignant Characteristics of Hepatocellular Carcinoma Cells by Inhibiting DNMT1 Expression. Front Pharmacol. 2020 Jun;11:867.
