# Supplementary material for: Polyphenols, aging, and health: What can we expect from the food industry in the technology era?
Source: Front Med (Lausanne). 2025 Nov 6;12:1671886. doi: 10.3389/fmed.2025.1671886 (PMC12630120; doi:10.3389/fmed.2025.1671886)
Supplement: Supplementary file 2 [file Table_2.docx]

**Supplementary material 2.** The effects of dietary polyphenols and their metabolites on aging-related diseases.

| **Polyphenol** | **Condition** | **Experimental design/trial** | **Main outcomes** | **Reference** |
| --- | --- | --- | --- | --- |
| Curcumin | Controlled type 2 DM, with 2- and 3-vessel coronary heart disease | Randomized, double-blind, placebo-controlled trial in 49 patients with controlled type 2 DM, aged 45 to 85 years, with 2- and 3-vessel coronary heart disease. Subjects (n=24) received 1000 mg/day curcumin or placebo (n=25) for 12 weeks | ↓ content of MDA  ↑ TAC and levels of GSH  ↑ PPAR-γ in peripheral blood mononuclear cells  did not affect mRNA expression for IL-1, IL-8, TGF-β, and VEGF | (1) |
| Meriva® (curcumin) | Individuals with a score ≥12 on the Australian Type 2 Diabetes Risk questionnaire | A 2x2 factorial, randomized, double-blind, placebo-controlled study in 29 diabetic patients aged 30–70 (years). The inclusion criteria were: diagnosed with impaired fasting glucose or impaired glucose tolerance or both; and HbA1c levels of 5.7–6.4%. Subjects in the test group received 180 mg curcumin/day for 12 weeks | ↓ circulating levels of GSK-3β  ↓ serum insulin and HOMA2-IR | (2) |
| Delphinol® | Impaired glucose tolerance | 43 healthy subjects (24 women, 19 men aged 19 to 50 years) presenting an abnormal glucose tolerance response (OGTT); one single dose of Delphinol (60, 120, and 180mg/capsule) or placebo at each experimental day with one-week washout period between each experimental day (open exploratory study) | ↓mean fasting glucose level (dose-dependent manner)  ↓ mean fasting insulin level (at higher dose)  ↔ Mean glycaemia and insulinemia variations during OGTT, using glucose as challenge. | (3) |
| Resveratrol (capsule) | Coronary artery disease | 10 patients (45 – 75 years) with coronary artery disease who have undergone percutaneous coronary intervention or post-myocardial revascularization undergoing optimal medical treatment and stable in terms of symptoms and pharmacotherapy for at least six weeks, intake of a capsule with 330 mg of resveratrol every 8 hours for 3 days. Medication taken by participants: antihypertensive medications ( n = 9); beta-blockers ( n = 6); antithrombotics ( n = 9); lipid-lowering ( n  = 9) and hypoglycemic ( n  = 2) medications. | ↓ FMD in patients after percutaneous coronary intervention  ↑ FMD in patients after coronary artery bypass grafting | (4) |
| Encapsulated (poly)phenol-rich aronia berry extract | Prehypertension | Randomized, double-blind, placebo-controlled trial. Prehypertensive volunteers, (SBP 120–139 mmHg and/or DBP 80–89 mmHg), men (n = 47) and women (n = 55), aged 40–70 years. Participants took 1 capsule of the product a day for 12 weeks. | ↑ arterial function in prehypertensive middle-aged individuals  ↔ blood pressure, endothelial function or blood lipids  ↑ health-promoting bacterial taxa (*I. butyriciproducens*, *B. faecihominis*, *B. xylanisolvens*). | (5) |
| Tablets of nutraceutical blend of artichoke and bergamot extracts | Metabolic and Vascular Risk Factors in Individuals with Suboptimal Cholesterol Levels | Randomized, double-blind, placebo-controlled, parallel-group in 90 adult volunteers with suboptimal cholesterol levels, aged 18 to 70 years.  LDL-C levels ranging from >115 mg/dL to <190 mg/dL, with triglyceride (TG) levels <400 mg/dL and an estimated 10-year cardiovascular risk of <5%.  Participants took 1 capsule of the product a day for 12 weeks. | ↑ serum lipids, systemic inflammation  ↑ indexes of NAFLD, and endothelial reactivity | (6) |
|  |  |  |  |  |
| Resveratrol (encapsulated) | Mild-moderate Alzheimer’s disease (AD) with CSF Aβ42 <600 ng/mL | Randomized, placebo-controlled, double-blind, retrospective study, multicenter 52-week phase 2 trial (aged 71.4 years, N = 36). Dosage: 500 mg/day, escalating by 500 mg every 13 weeks, up to 1000 mg BID. | ↓ cerebrospinal fluid - MMP9  ↑ MDC, IL-4, MMP10, and FGF-2  ↓ IL-12P40, IL12P70, and RANTES  modulated neuro-inflammation. | Moussa et al. (2017) |
| Coffee cherry extract (CCE) | Adults with mild cognitive decline without metabolic changes | Randomized, double blind, placebo-controlled, 28-day regimen (N=71, aged 55–65 years, BMI 20–28 kg/m², MoCA 18–25). Randomization into 4 groups: 1) 100 mg placebo BID (“Placebo-Placebo”); 2) 100 mg CCE in the morning and 100 mg placebo in the evening (“CCE-Placebo”); 3) 100 mg placebo in the morning and 100 mg CCE in the evening (“Placebo-CCE”); 4) 100 mg CCE BID (“CCE-CCE”). Short- and long-term cognitive challenges involving working memory processes were tested. | CCE, when taken in the morning or twice daily (BID), is associated with improvements in reaction times and trends toward indications of improved accuracy. | Robinson et al. (2020) |
| *Melissa officinalis* extract containing rosmarinic acid (RA) | Patients with mild dementia due to  Alzheimer’s disease | Randomized double-blind placebo-controlled parallel-group 24-week trial period (part 1), followed by an extension period of 24 weeks (part 2). In part 2, all patients were allocated to the M. officinalis group. (n = 23, mean age: 73 years, Dosage: 500 mg daily). | ↔ vital signs or physical and neurologic examination  ↔ cognitive measures  ↔ disease-related biomarkers  *M. officinalis* extract taken daily was safe and well-tolerated. | Noguchi-Shinohara et al. (2020) |
| Genistein aglicon | Postmenopausal and glucocorticoid treatment | 173 women aged 50 - 75 years and divided into genistein group (n=86) =2 tablets (27 mg of 98% pure genistein + 500 mg Calcium + 200 IU Vit. D3)/day 6 days/week and once a week 1 tablet (500 mg Calcium + 200 IU Vit. D3), (n=); group B (500 mg Calcium + 200 IU Vit. D3) or alendronate group (n=87) =2 tablets (500 mg Calcium + 200 IU Vit. D3)/day 6 days/week and once a week 1 tablet (70 mg alendronate), during 24 months in a randomized double-blind alendronate-controlled trial | ↔ body BMD  ↑ BMD anteroposterior lumbar spine, femoral neck, and total hip (both groups)  ↑osteocalcin and sclerostin (alendronate group)  ↓ osteocalcin and sclerostin (genistein group)  ↓ CTX, PTH (both groups)  ↑Bone ALP (both groups) | (11) |
| Equol and resveratrol | Postmenopausal and no hormone replacement therapy | 60 women aged 50 - 55 years and divided into treatment group (n=30) (200 mg of fermented soy dietary supplement containing 80 mg of isoflavone aglycones and 10 mg of equol + 25 mg of resveratrol) or placebo group (n=30); treatment during 12 months in a randomized, double-blind, placebo-controlled trial | ↑ body BMD  ↑ DPD, osteocalcin, and Bone-ALP  ↔ TRACP-5b | (12) |
| Freeze-dried blueberry powder | Postmenopausal (>4 years or total hysterectomy) | 13 women aged 51 - 61 years and divided into  low (17.5 g/d), medium (35 g/d), or high (70 g/d) dose of freeze-dried blueberry powder  equivalent to 0.75, 1.5, or 3 cups of fresh blueberries; treatment with a 6 weeks baseline period and 3 intervention periods followed by washout periods, each lasting for 6 weeks in a double-blind, crossover, randomized trial | ↔ serum calcium and 25(OH)D  ↔ bone resorption markers (CTX, NTx); osteocyte activity markers (osteoprotegerin, sclerostin); hormones (IGF-1, IGFBP-3)  ↓ bone resorption agonist RANKL (medium dose)  low and medium doses of blueberry for 6 weeks: greater bone calcium retention; high dose of blueberry: bone calcium retention was lower than in periods without intervention  ↔ urinary NTx normalized to creatinine | (13) |
| Prunes | Some degree of bone loss (BMD; t-score between 0.1 and ± 2.5 SD) | 35 men aged 55 – 80 years and divided into 100 g prunes/day (n=15), 50 g prunes/day (n=12), or control (n=8); all groups received 800 IU vitamin D and 450 mg calcium daily during 3 months in a randomized trial | ↑ body BMD  ↔ CPR, GPx  ↓ OPG (50 g prune group and control groups)  ↔ RANKL (50 g and 100g prune groups) ↓ control group  ↓ OPG: RANKL (50 g prune group)  ↓ osteocalcin | (14) |
| Epigallocatechin-3-Gallate | radiation-induced dermatitis (RID) in patients with breast cancer | Phase 2 double-blind, placebo-controlled randomized clinical trial enrolled 180 patients with breast cancer receiving postoperative radiotherapy.  Participants were randomly assigned (2:1) to receive either EGCG solution (660 μmol/L) or placebo (0.9% NaCl saline) sprayed to the whole radiation field from day 1 of the radiation until 2 weeks after radiation completion. | ↓ RID-related symptoms in patients with breast cancer receiving adjuvant radiotherapy | (15) |
| Green tea (GTE) and quercetin (Q) | Prostate cancer | Prospective, randomized, parallel design, placebo-controlled study enrolled 31 men with prostate cancer. Patients consumed daily 1 gram of GTE (830 mg of GTP) with 800 mg of Q (GT + Q) or placebo (GT + PL) for 4 weeks before prostatectomy for 3 weeks. | ↔ EGCG and ECG concentrations  ↔ decrease in methylation activity in prostate tissue or red blood cells.  No liver toxicity was observed | (16) |
| Green tea extract (GTE) | Colorectal adenomas | Single-center prospective randomized open-labelled trial in 186 patients with metachronous colorectal polyps among patients who underwent endoscopic removal of colorectal adenomas, aged 19 to 85 years old. Patients were randomized into 2 groups: supplementation group (0.9 g GTE per day for 12 months) or control group without GTE supplementation. The 72-h recall method was used to collect data on food items consumed by participants at baseline and the 1-year follow-up during the past 48 h. Follcolonoscopy was conducted 12 months later in 143 patients (71 in control group and 72 in the GTE group) | ↓ relapsed adenoma  ↔ BMI, serum lipid profiles, fasting serum glucose, and serum C-reactive protein levels  The study suggests a favorable outcome for the chemoprevention of metachronous colorectal adenomas | (17) |

**Symbol:** ↔ sample did not affect the parameter; ↓ sample induced significant reduction; ↑ sample induced significant increasing. **BMD,** bone mineral density; **BMI,** body mass index; **Bone-ALP,** Bone-specific alkaline phosphatase; **CRP**, C-reactive protein; **hs-CRP,** high‐sensitivity C‐reactive protein; **CTX**, C-terminal telopeptide type-1 collagen; **DPD**, deoxypyridinoline; **ECG;**  epicatechin gallate; **EGCG,**  epigallocatechin gallate; **FGF-2,** fibroblast growth factor; **FMD,** flow-mediated dilation; **GO,** glyoxal; **GPx,** glutathione peroxidase; **GSH,** reduced glutathione; **GSK-3β,** glycogen synthase kinase –β; **HDL,** high-density lipoproteins; **HDL-c,** high-density cholesterol; **HOMA2-IR,** insulin resistance; **IAPP,** islet amyloid polypeptide; **IGF-1**, insulin-like growth factor 1; **IGFBP-3**, insulin like growth factor-binding protein 3; **IL-1**, interleukin -1; **IL-4,** interleukin-4; **IL-8,** interleukin -8; **LDL,** low density lipoproteins; **LDL-c,** low‐density lipoprotein cholesterol; **MDA,** malondialdehyde; **MDC,** macrophage-derived chemokine; **MGO,** methylglyoxal; **MMP9,** human matrix metalloproteinase-9; **NTx**, N-terminal telopeptide; **OPG**, osteoprotegerin; **OGTT,** Oral Glucose Tolerance Test; **PPAR-γ,** peroxisome proliferator-activated receptor gamma; **PTH**, parathyroid hormone; **RANKL,** receptor activatorof NF-κB ligand; **RANTES,** regulated on activation, normal T-cell expressed and secreted; **SD**, Standard Deviation; **TAC,** total antioxidant capacity; **T2DM,** type 2 diabetes; **TGF-β,** transforming growth factor beta; **TNF-α,** tumor necrosis factor‐alpha; **TRACP-5b,** tartrate-resistant acid phosphatase 5b; **VEGF,** vascular endothelial growth factor.

Samples:

Curcumin capsules (Dineh Pharmaceutical Company, Tehran, Iran).

Delphinol® capsules (Barnafi Krause Farmacéutica S.A., Santiago, Chile) contain standardized maqui berry (*Aristotelia chilensis*) extract. Each 180mg/capsule contained 31.5% delphinidin glycosides and 39.4% total anthocyanins.

Fermented soy dietary supplement (Equopausa D. Ulrich, Paladin Pharma S.p.A., Turin, Italy

Genistein aglicon (Primus Pharmaceuticals Inc., Scottsdale, AZ, USA).

Meriva®, capsules of Curcumin phospholipids (Indena SpA, Milan, Italy) from *Curcuma longa* L. Each capsule contains 18-22% total curcuminoids.

Resveratrol (98.57% pure, *Polygonum cuspidatum* extract; microcrystalline cellulose, 21st Century Alternatives, GB), capsules of 330 mg resveratrol each.

**Reference**

1. Shafabakhsh R, Mobini M, Raygan F, Aghadavod E, Ostadmohammadi V, Amirani E, et al. Curcumin administration and the effects on psychological status and markers of inflammation and oxidative damage in patients with type 2 diabetes and coronary heart disease. Clin Nutr ESPEN. 2020;40:77–82.

2. Thota RN, Rosato JI, Dias CB, Burrows TL, Martins RN, Garg ML. Dietary Supplementation with Curcumin Reduce Circulating Levels of Glycogen Synthase Kinase-3β and Islet Amyloid Polypeptide in Adults with High Risk of Type 2 Diabetes and Alzheimer’s Disease. Nutrients [Internet]. 2020 Apr 9;12(4):1032. Available from: https://www.mdpi.com/2072-6643/12/4/1032

3. Alvarado JL, Leschot A, Olivera-Nappa Á, Salgado A-M, Rioseco H, Lyon C, et al. Delphinidin-Rich Maqui Berry Extract (Delphinol®) Lowers Fasting and Postprandial Glycemia and Insulinemia in Prediabetic Individuals during Oral Glucose Tolerance Tests. Biochem Res Int. 2016;

4. Diaz M, Avila A, Degens H, Coeckelberghs E, Vanhees L, Cornelissen V, et al. Acute resveratrol supplementation in coronary artery disease: towards patient stratification. Control Clin Trial [Internet]. 2020;54(1):14–9. Available from: https://pubmed.ncbi.nlm.nih.gov/31429599/

5. Le Sayec M, Xu Y, Laiola M, Gallego FA, Katsikioti D, Durbidge C, et al. The effects of Aronia berry (poly)phenol supplementation on arterial function and the gut microbiome in middle aged men and women: Results from a randomized controlled trial. Clin Nutr. 2022;41(11).

6. Fogacci F, Giovannini M, Di Micoli A, Fiorini G, Grandi E, Borghi C, et al. A Randomized, Double-Blind, Placebo-Controlled Clinical Trial on the Effect of a Dietary Supplement Containing Dry Artichoke and Bergamot Extracts on Metabolic and Vascular Risk Factors in Individuals with Suboptimal Cholesterol Levels. Nutrients [Internet]. 2024 May 23;16(11):1587. Available from: https://www.mdpi.com/2072-6643/16/11/1587

7. Morris MC, Wang Y, Barnes LL, Bennett DA, Dawson-Hughes B, Booth SL. Nutrients and bioactives in green leafy vegetables and cognitive decline. Neurology [Internet]. 2018 Jan 16;90(3). Available from: https://www.neurology.org/doi/10.1212/WNL.0000000000004815

8. Moussa C, Hebron M, Huang X, Ahn J, Rissman RA, Aisen PS, et al. Resveratrol regulates neuro-inflammation and induces adaptive immunity in Alzheimer’s disease. J Neuroinflammation. 2017;14(1):1–10.

9. Robinson JL, Hunter JM, Reyes-Izquierdo T, Argumedo R, Brizuela-Bastien J, Keller R, et al. Cognitive short- and long-term effects of coffee cherry extract in older adults with mild cognitive decline. Aging, Neuropsychol Cogn [Internet]. 2020 Nov 1;27(6):918–34. Available from: https://www.tandfonline.com/doi/full/10.1080/13825585.2019.1702622

10. Noguchi-Shinohara M, Ono K, Hamaguchi T, Nagai T, Kobayashi S, Komatsu J, et al. Safety and efficacy of Melissa officinalis extract containing rosmarinic acid in the prevention of Alzheimer’s disease progression. Sci Rep [Internet]. 2020 Oct 29;10(1):18627. Available from: https://www.nature.com/articles/s41598-020-73729-2

11. Squadrito F, Imbalzano E, Rottura M, Arcoraci V, Pallio G, Catalano A, et al. Effects of genistein aglycone in glucocorticoid induced osteoporosis: A randomized clinical trial in comparison with alendronate. Biomed Pharmacother [Internet]. 2023 Jul;163:114821. Available from: https://linkinghub.elsevier.com/retrieve/pii/S075333222300611X

12. Corbi G, Nobile V, Conti V, Cannavo A, Sorrenti V, Medoro A, et al. Equol and Resveratrol Improve Bone Turnover Biomarkers in Postmenopausal Women: A Clinical Trial. Int J Mol Sci [Internet]. 2023 Jul 27;24(15):12063. Available from: https://www.mdpi.com/1422-0067/24/15/12063

13. Hodges JK, Maiz M, Cao S, Lachcik PJ, Peacock M, McCabe GP, et al. Moderate consumption of freeze-dried blueberry powder increased net bone calcium retention compared with no treatment in healthy postmenopausal women: a randomized crossover trial. Am J Clin Nutr [Internet]. 2023 Aug;118(2):382–90. Available from: https://linkinghub.elsevier.com/retrieve/pii/S0002916523659611

14. George KS, Munoz J, Ormsbee LT, Akhavan NS, Foley EM, Siebert SC, et al. The Short-Term Effect of Prunes in Improving Bone in Men. Nutrients [Internet]. 2022 Jan 10;14(2):276. Available from: https://www.mdpi.com/2072-6643/14/2/276

15. Zhao H, Zhu W, Zhao X, Li X, Zhou Z, Zheng M, et al. Efficacy of Epigallocatechin-3-Gallate in Preventing Dermatitis in Patients With Breast Cancer Receiving Postoperative Radiotherapy. JAMA Dermatology [Internet]. 2022 Jul 1;158(7):779. Available from: https://jamanetwork.com/journals/jamadermatology/fullarticle/2792713

16. Henning SM, Wang P, Lee R-P, Trang A, Husari G, Yang J, et al. Prospective randomized trial evaluating blood and prostate tissue concentrations of green tea polyphenols and quercetin in men with prostate cancer. Food Funct [Internet]. 2020;11(5):4114–22. Available from: https://xlink.rsc.org/?DOI=D0FO00565G

17. Shin CM, Lee DH, Seo AY, Lee HJ, Kim SB, Son W-C, et al. Green tea extracts for the prevention of metachronous colorectal polyps among patients who underwent endoscopic removal of colorectal adenomas: A randomized clinical trial. Clin Nutr [Internet]. 2018 Apr;37(2):452–8. Available from: https://linkinghub.elsevier.com/retrieve/pii/S0261561417300389
